# Identification and Characterization of a Small-Molecule Inhibitor of the *Pseudomonas aeruginosa* SOS Response

**DOI:** 10.1021/acsinfecdis.5c00467

**Published:** 2025-11-07

**Authors:** Filippo Vascon, Benedetta Fongaro, Vytautas Mickevičius, Antonella Pasquato, Birute Grybaite, Vidmantas Petraitis, Rahma Ben Abderrazek, Patrizia Polverino de Laureto, Donatella Tondi, Povilas Kavaliauskas, Laura Cendron

**Affiliations:** † Department of Biology, University of Padova, Via Ugo Bassi 58/b, Padova 35131, Italy; ‡ Department of Pharmaceutical and Pharmacological Sciences, University of Padova, Via F. Marzolo 5, Padova 35131, Italy; § Department of Organic Chemistry, 70309 Kaunas University of Technology, Radvilenu Rd. 19, Kaunas LT-50254, Lithuania; ∥ Department of Surgery, Oncology and Gastroenterology, University of Padova, Via Giustiniani 2, Padova 35128, Italy; ⊥ Center for Discovery and Innovation, Hackensack Meridian Health, Nutley, New Jersey 07110, United States of America; # Laboratoire des Biomolécules, Venins et Applications Théranostiques, Institut Pasteur Tunis, 37964 Université Tunis El Manar, 13 Place Pasteur, Tunis 2092, Tunisia; ¶ Department of Life Sciences, University of Modena and Reggio Emilia, Via Campi 103, Modena 41125, Italy; ∇ Department of Microbiology and Immunology, University of Maryland School of Medicine, Baltimore, Maryland 21201, United States

**Keywords:** SOS response, *Pseudomonas aeruginosa*, RecA, LexA, antimicrobial resistance, virulence

## Abstract

The SOS response is among the most conserved pathways that promote antibiotic resistance onset in bacteria. This study aimed to identify and characterize small-molecule inhibitors of the SOS response in the opportunistic pathogen *Pseudomonas aeruginosa*. A library of 318 drug-like compounds was screened for inhibition of RecA-induced LexA autoproteolysis, a key step in SOS activation. One hit compound, 3-(2-sulfanylanilino)­propanoic acid, showed dose-dependent inhibition with an IC_50_ in the mid-micromolar. Differential scanning fluorimetry and isothermal titration calorimetry revealed that A12 binds to both RecA and LexA with a low micromolar affinity. Mass spectrometry analysis demonstrated that A12 covalently modifies RecA via condensation, while it forms a disulfide bond with Cys104 of LexA. Inhibition was diminished under reducing conditions, confirming that disulfide formation is crucial for A12 activity. A12 did not impair LexA’s ability to bind SOS box DNA sequences, which is needed to keep the SOS genes repressed. Antimicrobial susceptibility testing of A12 on *P. aeruginosa* PAO1 showed no additive effect with tested antibiotics, but it impaired *P. aeruginosa* colonization of A549 lung epithelial cells and survival in THP-1-derived macrophages. While A12’s potency requires optimization, it represents a promising scaffold for developing anti-SOS compounds targeting *P. aeruginosa*.

## Graphical abstract

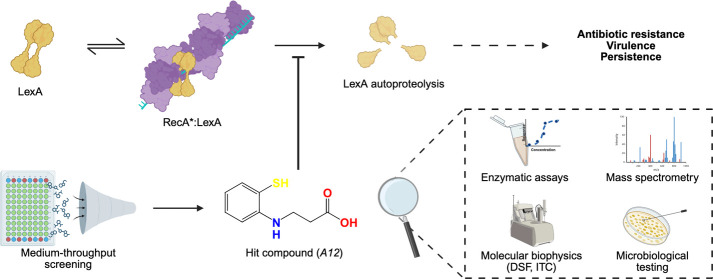

*Pseudomonas aeruginosa* is an opportunistic bacterial pathogen of high clinical relevance, characterized by a broad spectrum of resistance to currently available antibiotics.^1^ Finding new therapeutic strategies that efficiently and selectively kill this microorganism or increase its sensitivity to antibiotics is, thus, a priority.

The bacterial SOS response to DNA damage—orchestrated by the DNA-damage sensor RecA and the autoproteolytic transcriptional repressor LexA (recently thoroughly reviewed by^2–4^ ; Figure 1A)—regulates DNA repair and mutagenesis, thus representing a driver of antibiotic resistance.^5–8^ Moreover, in *P. aeruginosa,* this pathway—also through other LexA-like transcriptional regulators^9–11^ —controls the expression of virulence factors^11,12^ and the formation of biofilms,^13^ so its inhibition could be greatly beneficial for the eradication of *P. aeruginosa* infections.

**Figure 1:**
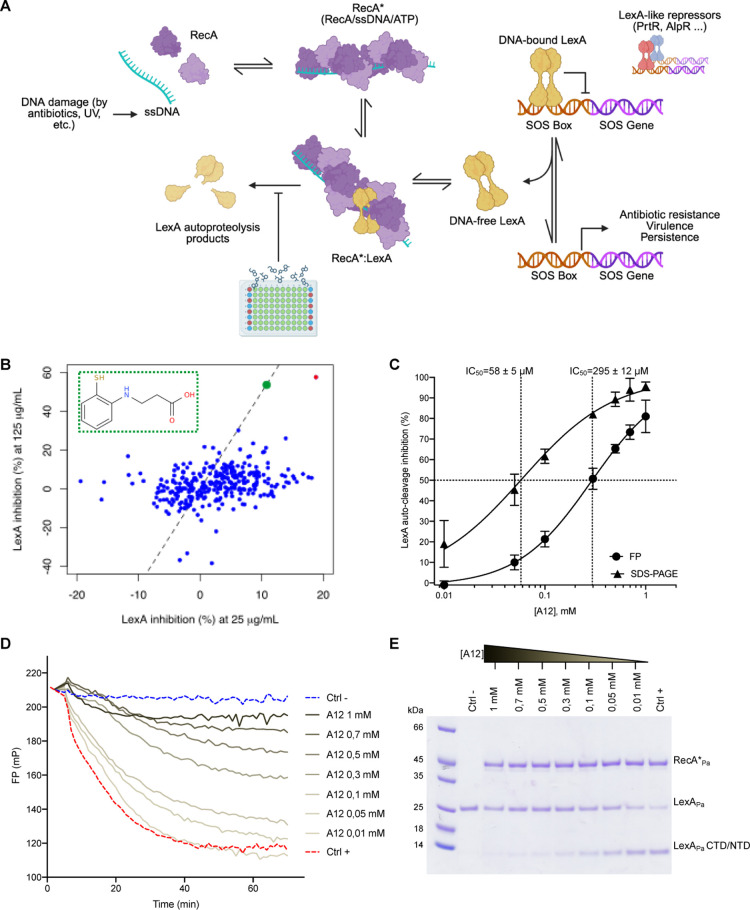
In vitro identification of a new inhibitor of *P. aeruginosa*SOS system. (A) Schematic representation of the bacterial SOS response to DNA damage. The RecA*-induced LexA autoproteolysis is the desired target of our screening campaign. (B) LexA autoproteolysis percent inhibition values obtained in the initial FP-based library screening at two concentrations (25 and 125 μg/mL). Among the two compounds showing a consistent activity at the two concentrations tested, one revealed to be a false positive (red dot), while compound A12 was a true positive (green dot and inset). The dotted line represents *y* = 5*x* function. (C) Dose–response curves of A12 on RecA*_Pa_-induced LexA_Pa_ autoproteolysis, as obtained by FP (one replicate shown in panel D) and SDS-PAGE (one representative gel shown in panel E) assays. Data points in panel C represent averages of 3 replicates ± standard deviation. Uncertainties on IC_50_ values correspond to the standard error.

We and others have recently solved the long-standing question on how the two SOS response master regulators, RecA and LexA, interact with each other, showing a high structural conservation between *E. coli* and *P. aeruginosa*.^14–16^

The druggability of the SOS response by small molecules and peptides has already been demonstrated,^17–26^ but no anti-SOS compounds have entered clinical testing. The SOS system presents several hurdles that have likely hindered the discovery of a potent inhibitor with drug-like properties, including: (i) the homology of RecA with the human Rad51 family, which calls for cautious off-target evaluation, (ii) the limited dimensions and strong substrate specificity of the LexA active site,^27^ which reduce the explorable chemical space of potential inhibitors, and (iii) the intramolecular nature of LexA proteolysis (the core of the SOS activation), which implies an effective local substrate concentration hard to be overwhelmed by ectopic competitive inhibitors.^28^

These challenges were recently overcome by anti-LexA nanobodies that target the LexA cleavage site and its conformational dynamics,^29^ but this approach still needs optimization, in particular in terms of delivery routes.

In this context, new high-throughput screenings of molecular fragments could provide promising scaffolds for suppressing the SOS response via previously unexplored pharmacophores and mechanisms of action.

The highest advantages of small, drug-like compounds over therapeutic peptides and biomolecules are mainly related to their membrane permeability, pharmacokinetic profile, and potential stability. In particular, their low molecular weight (<1 kDa) and usually high hydrophobicity allow them to easily diffuse through biological membranes, reaching cytoplasmic targets such as RecA and LexA in bacteria.

The objective of studies reported in this work is to identify and characterize potential inhibitors of *P. aeruginosa* SOS response among a library of drug-like compounds, which has been previously screened and provided inhibitors of proteolytic enzymes, such as Furin.^30^

A hit compound (hereafter referred to as A12) emerged from the primary library screening and its effect on the *P. aeruginosa* SOS system (RecA_Pa_ and LexA_Pa_) was extensively analyzed in vitro by LexA autoproteolysis inhibition assays, differential scanning fluorimetry (DSF), isothermal titration calorimetry (ITC), electrophoretic mobility shift assay (EMSA), and mass spectrometry (MS). A12 confirmed the ability to inhibit LexA_Pa_ self-cleavage promoted by activated RecA_Pa_ (RecA_Pa_/ssDNA/ATPγS, RecA_Pa_*), with an IC_50_ in the mid-micromolar range. Mechanistic dissection evidenced a dual targeting that requires further elucidation and the examination of A12-based libraries of compounds that explore a broader chemical space.

## Results

### Screening of a Library of Small Molecules and Validation of Selected Hits

A custom-made library of 318 small molecules (MW < 1000 Da) has been screened at two different concentrations (25 μg/mL and 125 μg/mL, corresponding to molar concentrations of 30–170 μM and 150–850 μM, respectively) on the *P. aeruginosa* SOS system by a previously validated fluorescence polμarization (FP)-based RecA*-induced LexA autoproteolysis in vitro assay.^16,20,21,29,31^ 1 μM FlAsH-LexA_Pa_^CTD^ and 1 μM preactivated RecA*_Pa_ were incubated with the screened chemical fragments for 1 h at 37 °C before measuring FP.

Two compounds (referred to as “A12” and “E17”) displayed an increase of LexA autoproteolysis inhibition reasonably consistent with the tested concentrations and exceeded 50% inhibition at 125 μg/mL (Figure 1B).

Then, the FP-based LexA autoproteolysis assay was repeated on the two hits, monitoring the full kinetics of LexA cleavage over a 1 h incubation and exploring a wide range of drug concentrations (10–1000 μM). Compound E17 proved to be a false positive, as it induced evident protein aggregation events (Figure S1), causing a very high FP increase after adding the compound to the protein mixture.

Conversely, A12 manifested a negligible protein precipitation effect and a clear dependence of LexA inhibition on drug concentration, reaching 80% of LexA self-cleavage suppression at 1 mM. Higher concentrations of A12 were not explored, since its solubility in aqueous buffers strongly worsened above 500 μM and would have misled any inhibition data.

An IC_50_ of 295 ± 12 μM was obtained from data fitting (Figure 1C,D). A12 was disclosed to be 3-(2-sulfanylanilino)­propanoic acid (structure in Figure 1B, inset).

To further validate the activity of A12 on RecA*_Pa_-LexA_Pa_, an assay was performed using full-length LexA_Pa_, and autoproteolysis was followed by SDS-PAGE in the presence of different concentrations of A12 (Figure 1E). Cleavage reactions were performed at 1 μM full-length LexA_Pa_, initiated by the addition of 1 μM RecA*_Pa_, and stopped after a 1 h incubation at 37 °C, corresponding to the same conditions used for the FP assay.

SDS-PAGE analysis of LexA_Pa_ autoproteolysis demonstrated a clear reduction of cleavage products (and a corresponding intensification of the band of uncleaved LexA_Pa_) at increasing concentrations of A12. Data fitting resulted in an IC_50_ of 58 ± 5 μM, which is 5 times lower than the value obtained in the FP assay. This discrepancy might result from intrinsic differences and limitations of the two techniques or from a higher affinity of A12 to the full-length LexA_Pa_ than its CTD domain alone.

### A12 Binding to SOS Response Players

To elucidate whether A12 binds RecA_Pa_ or LexA_Pa_, the two proteins were submitted to a thermal shift assay by DSF, in the presence of different concentrations of A12 (30–1000 μM; Figure 2A–C). Controls devoid of A12 were included, as well. To avoid any potential self-cleavage of LexA_Pa_, the S125A inactive mutant was used.

**Figure 2:**
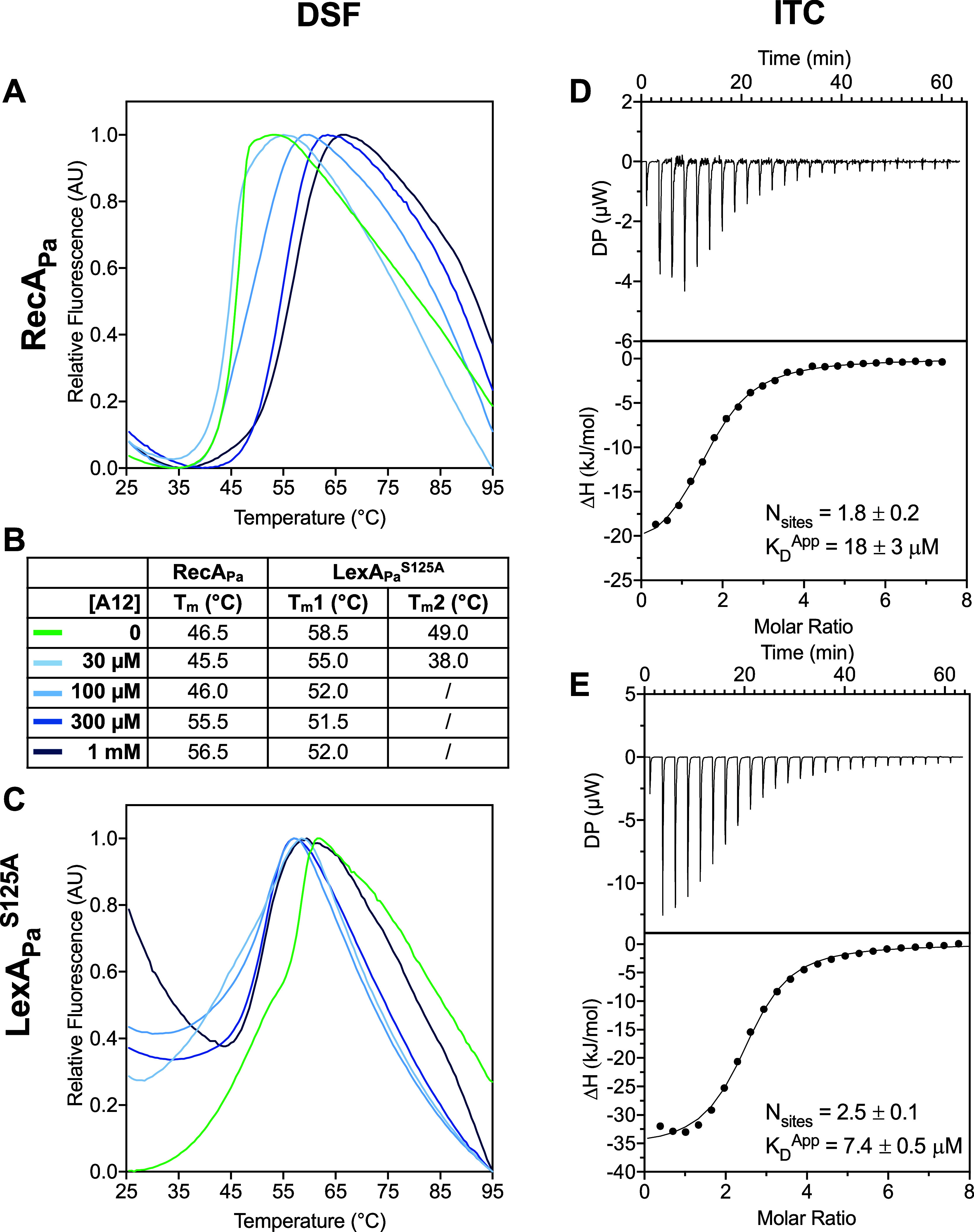
Analysis of A12 binding to RecA_Pa_ and LexA_Pa_^S125A^ by DSF and ITC. (A,C) Normalized melting curves of RecA_Pa_ and LexA_Pa_^S125A^ in the presence of different concentrations of A12. One representative of two coherent replicates is reported. (B) Melting temperatures, corresponding to local or global minima of melting curve derivatives. (D,E) Raw traces (top) and integrated binding isotherms (bottom) of ITC analysis of A12 on purified RecA_Pa_ and LexA_Pa_^S125A^ at 298 K. Integrated heats were fitted by a ≪one set of sites≫ model. One representative curve is shown for each condition. The number of equivalent binding sites (*N*_sites_) and the apparent equilibrium dissociation constant (*K*_D_) are represented as average ±S.D., calculated on at least two replicates.

The sharp melting curve of RecA_Pa_ (*T*_m_ 46.5 °C) was noticeably shifted rightward by increasing concentrations of A12, with a significant Δ*T*_m_ of 10 °C at 1000 μM A12 compared to the control (Figure 2A,B), a clear indication of A12 binding to RecA_Pa_.

Concerning full-length LexA_Pa_^S125A^, a biphasic melting curve was obtained for the pure protein (*T*_m1_ = 49.0 °C; *T*_m2_ = 58.5 °C; Figure 2C), most likely due to the independent denaturation of its two domains (the DNA-binding NTD and the autoproteolytic CTD) assembled into dimers. Treatment with A12 at increasing concentrations induces some unexpected events on LexA_Pa_^S125A^: at 30 μM, A12 seems to cause a general destabilization of LexA_Pa_^S125A^, as both the melting transitions are shifted toward lower temperatures, while in the presence of A12 above 100 μM monophasic melting curves are obtained, with a flexus between *T*_m1_ and *T*_m2_ (at 52.0 °C; Figure 2B). This behavior suggests that A12 induces some conformational rearrangement on LexA_Pa_^S125A^, probably interacting with a region at the boundary between the two domains or close to the dimerization interface.

To investigate deeper the binding of A12 to the two members of the *P. aeruginosa* SOS complex, ITC was performed on RecA_Pa_ and LexA_Pa_^S125A^ (Figure 2D,E). The integrated binding isotherms of both proteins were best fitted by a “one set of sites” model. Roughly two equivalent binding sites were detected on each protein, while the affinity of A12 for LexA_Pa_^S125A^ (*K*_D_ = 7.4 ± 0.5 μM) was slightly higher than that for RecA_Pa_ (*K*_D_ = 18 ± 3 μM). Taken together, DSF and ITC data suggest that A12 is able to bind both the SOS response control proteins with micromolar affinity.

### A12 Inhibition on LexA_Pa_ Requires Disulfide-Mediated Covalent Binding

Since A12, 3-(2-sulfanylanilino)­propanoic acid, is characterized by the presence of a thiol group and both RecA_Pa_ and LexA_Pa_ have some cysteine residues not involved in intramolecular disulfide bridges, the possibility that A12 targets the SOS system via the formation of disulfides was investigated. Moreover, we considered that in the experimental conditions used throughout this work, A12 could form a disulfide-mediated adduct, thus converting back to its synthesis intermediate 3-[2-[[2-(2-carboxyethylamino)­phenyl]­disulfanyl]­anilino]­propanoic acid, which might represent the biologically active form. These possibilities were investigated by MS and fluorescence polarization assays (FP).

First, ESI-MS analysis of A12 (2 mM, diluted in the same buffer used for FP assays) revealed a predominant species with a molecular weight of 394.17 ± 0.05 Da (Figure 3A), which is compatible with that of 3-[2-[[2-(2-carboxyethylamino)­phenyl]­disulfanyl]­anilino]­propanoic acid (392.5 Da). The same analysis was conducted after treatment of A12 with 2 mM DTT (Figure 3B). In this sample, the MS signals corresponding to the disulfide adduct were absent, leading to the conclusion that the dimerization of A12 by the formation of a disulfide bridge can occur under physiological conditions. When in monomeric form, the compound is likely degraded and/or not detectable by MS under the tested experimental conditions.

**Figure 3:**
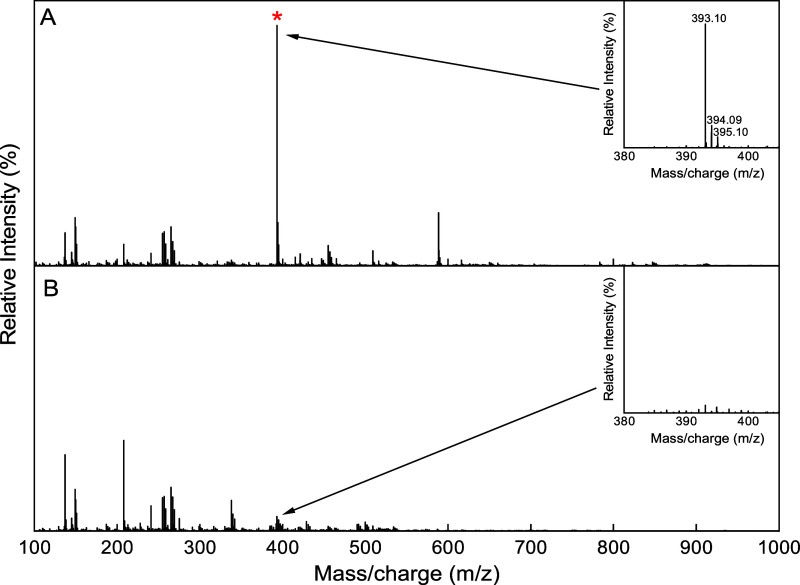
Positive-ion ESI mass spectrum of A12. (A) A12 in the absence of DTT, showing a dimeric form of the compound (red star). Inset: zoom of the region showing the charge distribution of the chemical compound. (B) A12 incubated with DTT (2 mM), showing the absence of the dimer in reducing conditions. Insert: zoom of the region previously containing the dimeric signal.

Then, ESI-MS analysis was carried out on both RecA_Pa_ and LexA_Pa_ alone and preincubated with A12 (referred to as RecA_Pa_-A12 and LexA_Pa_-A12, respectively).

Compared to the unreacted control (which displayed a single protein species; Figures 4A and S2A), the mass spectrum of RecA_Pa_-A12 showed the presence of two protein species (Figures 4B and S2A) with a 179 Da mass difference. This difference is compatible with a condensation reaction between A12 and RecA_Pa_, with the release of a molecule of water. A tryptic digestion of RecA_Pa_-A12 was submitted to LC–MS analysis (Figure S2A,B), revealing that the chemical modification is located on the 270–296 fragment of RecA_Pa_ (TGEIIDLGVQLGLVEKSGAWYSYQGSK; residues 285–311 in the 6xHis-tagged protein). The modified peptide has a molecular mass of 3076.55 ± 0.22 Da, 179 Da higher than the expected MW for the native one. Tandem mass analysis revealed the presence of an A12-modified lysine at position 285, strongly supporting an amide bond formation between the A12 carboxylate and Lys285 ε-amino group.

**Figure 4:**
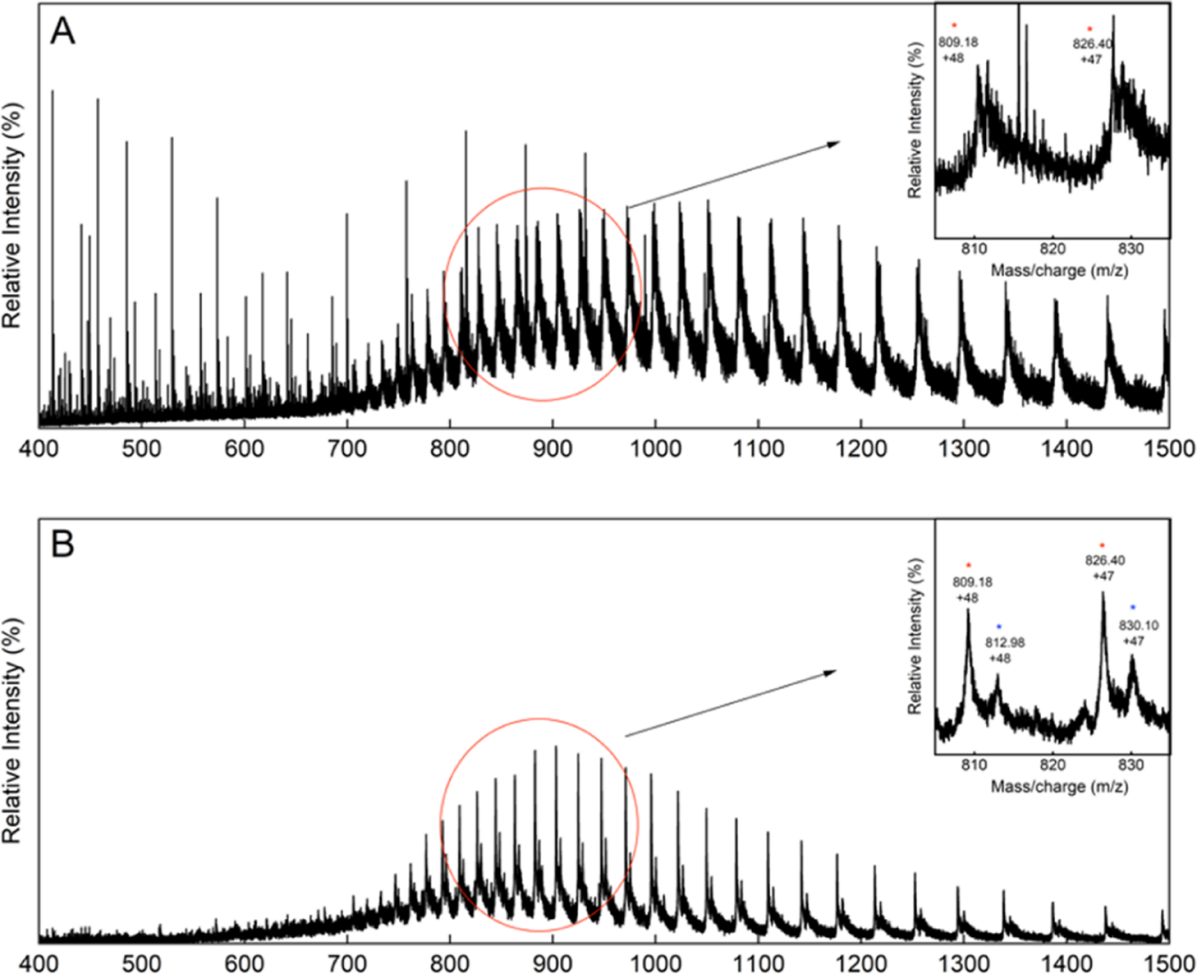
MS analysis of RecA_Pa_ incubated with A12. (A) Mass spectrum of RecA_Pa_. (B) Mass spectrum of RecA_Pa_-A12, showing the appearance of a second mass distribution. Insets: zoom of the range 800–900 *m*/*z*, showing the signals of the protein distribution, corresponding to RecA_Pa_ (red star) and to the A12-bound RecA_Pa_ (blue star), with a mass delta of 179 Da.

The mass spectrum of LexA_Pa_ (Figures 5A and S2A) shows a single species corresponding to the unmodified protein. Conversely, MS analysis of LexA_Pa_-A12 revealed the presence of two protein species: one with the expected molecular weight for LexA_Pa_ (Figures 5B, red star; S2A) and the other with a molecular weight 195 Da higher (Figure 5B, blue star). The latter probably corresponds to LexA_Pa_ covalently bound by A12 by a disulfide bond, as a 2 Da mass difference between A12 and the identified modification is compatible with thiol deprotonation upon disulfide formation.

**Figure 5:**
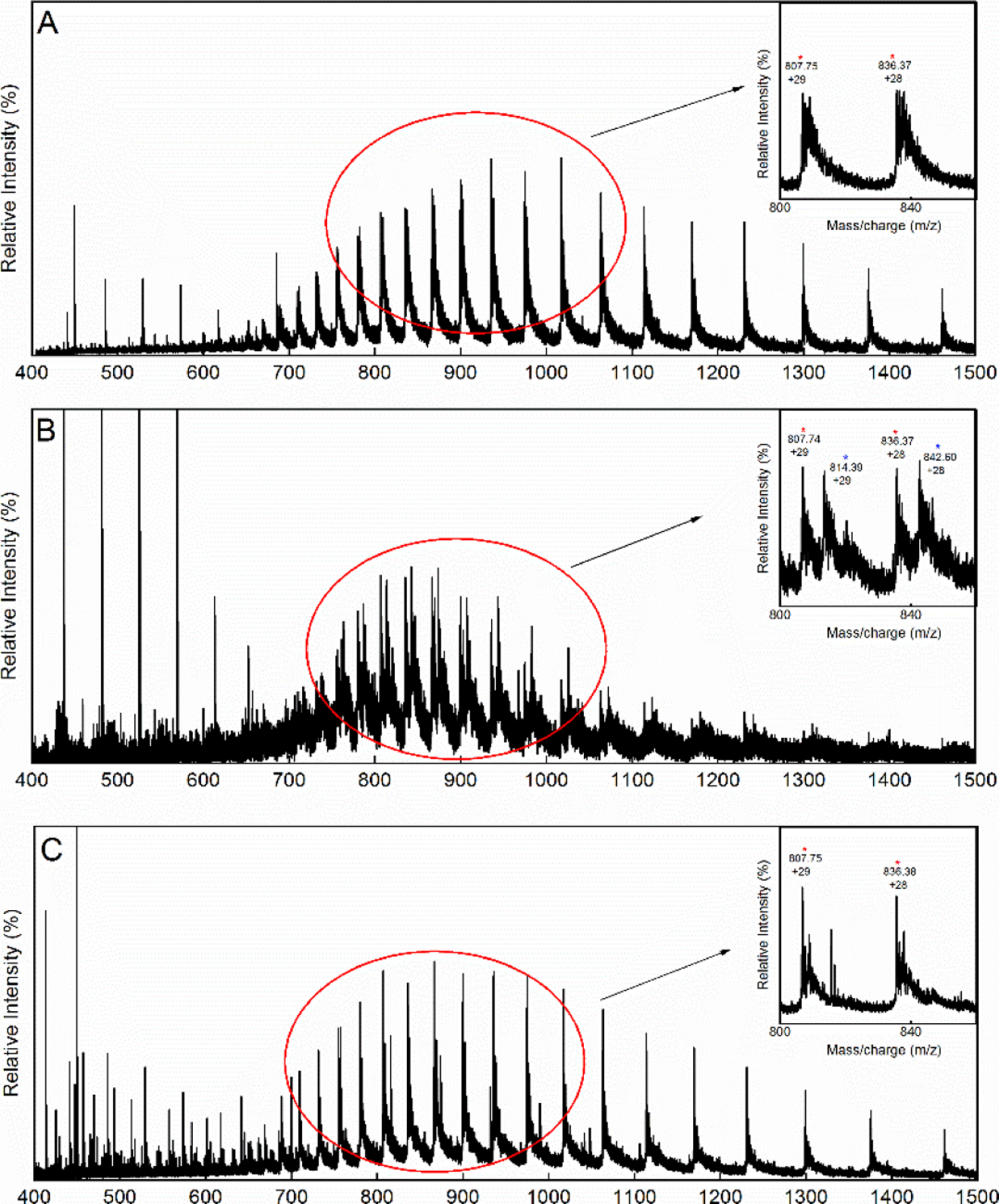
MS analysis of LexA_Pa_ incubated with A12. (A) Mass spectrum of LexA_Pa_. (B) Mass spectrum of LexA_Pa_-A12, showing the appearance of a second mass distribution. (C) Mass spectrum of LexA_Pa_-A12 treated with DTT, showing that the chemical modification is lost after the reducing treatment. Insets: zoom of the 800–900 *m*/*z* range, showing the signals of the protein distribution without (red star) and with (blue star) a delta mass of 195 Da.

A control analysis was performed in the presence of 5 mM DTT, and the chemically modified LexA_Pa_ was absent (Figures 5C and S2A and C), confirming that A12 covalent binding on LexA_Pa_ relies on an oxidation reaction.

Once again, tryptic digestion and comparative LC–MS analysis of LexA_Pa_ alone and coincubated with A12 allowed the identification of the chemically modified peptide (Figure S2B), corresponding to the 88–105 fragment (VAAGAPILAEQNIEESCR; residues 95–112 considering the 6xHisTag), which includes Cys104 (Cys111 in the 6xHis-tagged protein). The unmodified peptide has a molecular mass of 1869.93 ± 0.08 Da, while the modified one showed an increase in mass of 195 Da. These data led to the conclusion that A12 is able to bind LexA_Pa_ by the formation of disulfide bond at the level of Cys104. As noted above, A12 appears mostly in a dimeric state in solution; however, it can still react with LexA_Pa_ to form a new disulfide bond via a thiol–disulfide exchange^32^ controlled by multiple equilibria (established between dimeric and monomeric A12 and between free monomeric A12 and the LexA_Pa_-A12 adduct).

We further investigated the relevance of the disulfide-mediated chemical binding of A12 to LexA_Pa_ by the FP-based autoproteolysis assay under reducing conditions (2 mM TCEP). The reducing environment significantly diminished A12 inhibitory activity compared to a nonreducing condition (Figure 6A), confirming that redox equilibria have an impact on its mechanism of action.

**Figure 6:**
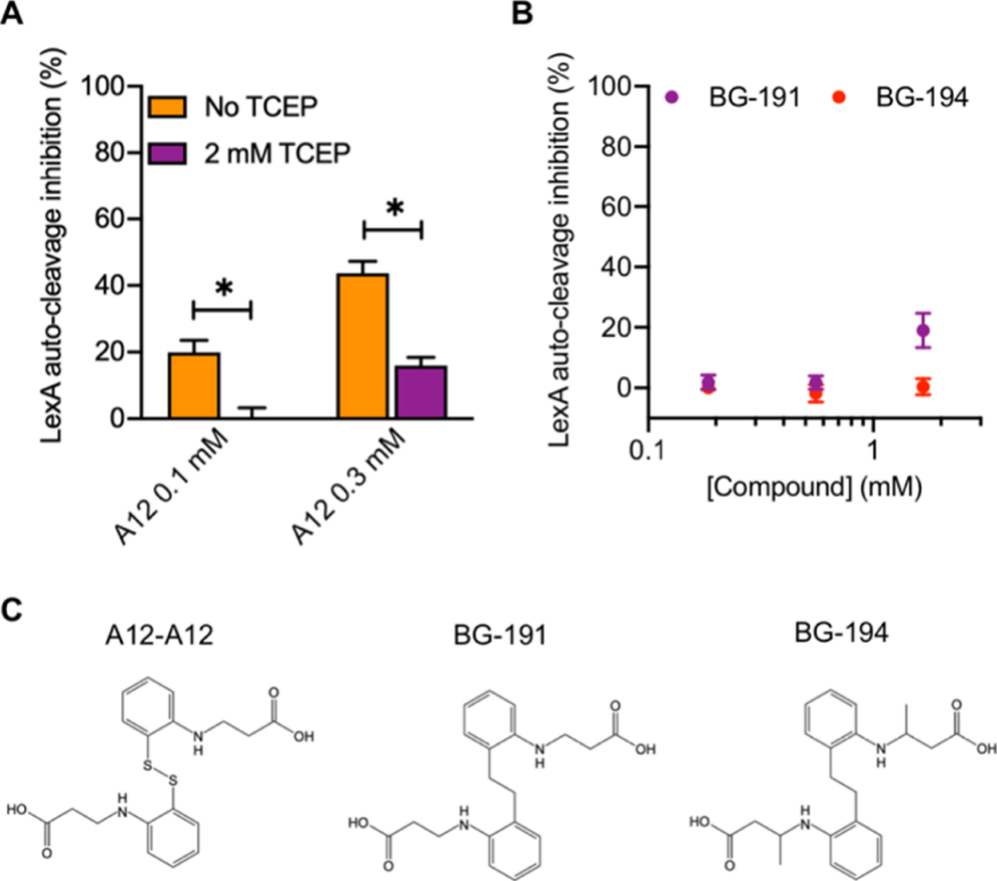
Activity testing of A12 in a reducing environment and of A12 derivatives. (A) LexA_Pa_ autocleavage percent inhibition obtained by the FP-based assay with different concentrations of A12, either in the presence or absence of a reducing agent (TCEP). (B) LexA_Pa_ autocleavage percent inhibition obtained by the FP-based LexA_Pa_ autoproteolysis assay with different concentrations of A12 derivatives. (C) Chemical structures of A12 covalent adduct and derivatives. Data represent the mean ± SEM of three replicates. *P-value <0.03.

To further exclude that the biologically active form of A12 is its covalent dimeric state, an analog corresponding to two A12 moieties devoid of sulfur atoms and linked by an ethylene arm (3,3′-((ethane-1,2-diylbis­(2,1-phenylene))­bis­(azanediyl))­dipropionic acid, referred to as BG-191) was synthesized and examined by the same FP-based LexA_Pa_ self-cleavage assay. A slightly modified congener (3,3′-((ethane-1,2-diylbis­(2,1-phenylene))­bis­(azanediyl))­dibutyric acid, referred to as BG-194) was tested as well. Both compounds displayed severely lowered activity compared to A12 in a range of concentrations between 0.2 and 2 mM. While BG-194 did not display any inhibitory activity on LexA_Pa_, BG-191 reached only 20% inhibition at the highest concentration tested (Figure 6B). Conversely, as discussed above, A12 almost completely prevented LexA_Pa_ autoproteolysis at 1 mM. Taken together, these observations suggest that the observed activity of A12 mostly relies on the direct binding of its monomeric form to LexA_Pa_ via disulfide bridging (Figure 7A,B). However, we cannot exclude that A12 covalent binding to RecA_Pa_ has an impact on its biological activities (including coprotease), despite likely being less relevant on the overall inhibition of the SOS system.

**Figure 7:**
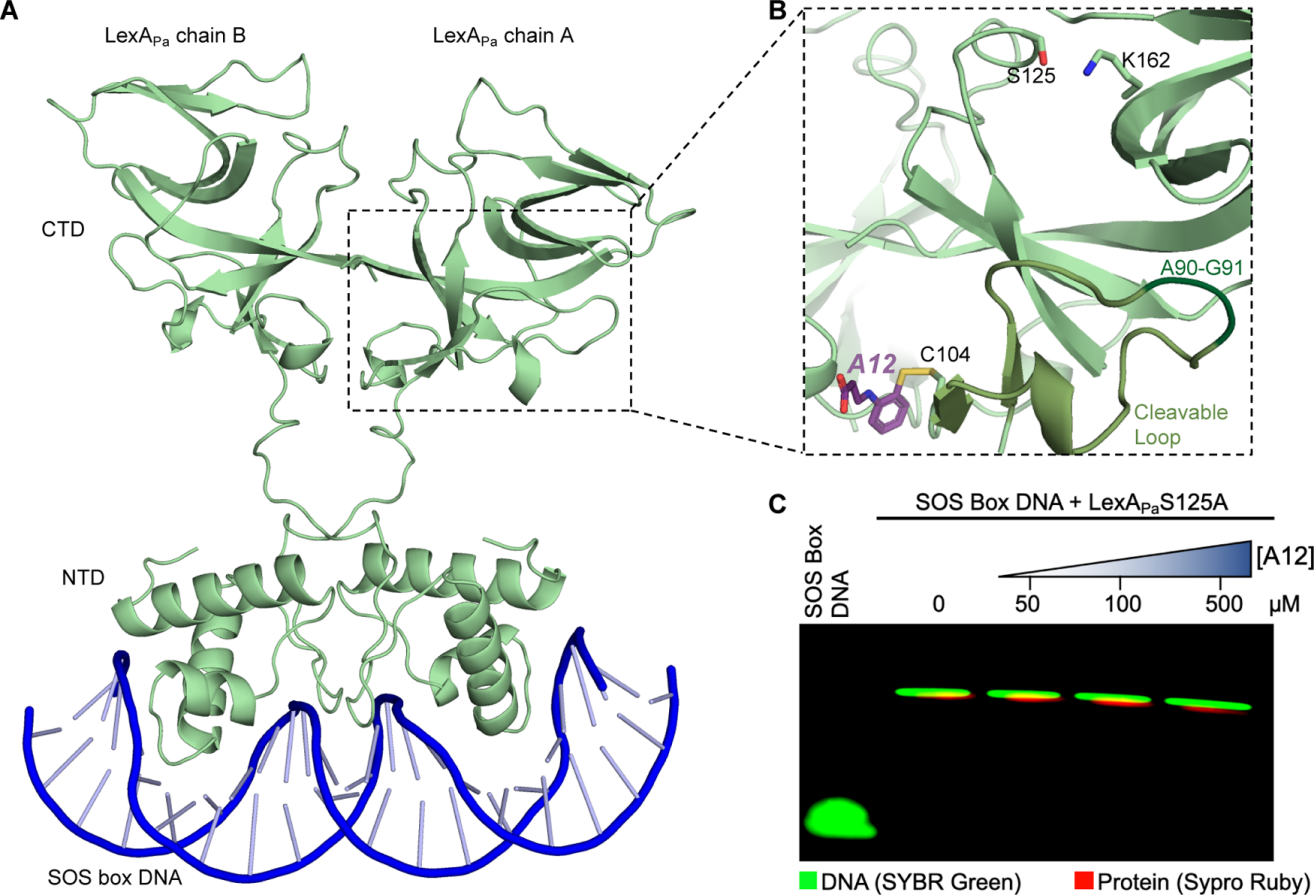
A12 binding site on LexA_Pa_. (A) AlphaFold3 model of full-length LexA_Pa_ bound to SOS box dsDNA. (B) Zoom on A12 binding site on LexA_Pa_^CTD^ (light green; PDB: 8B0V)_,_ at the base of the cleavable loop (medium green; the cleavage site A90-G91 is depicted in dark green) and close to the dimerization interface. The catalytic dyad S125/K162 is shown as sticks. (C) EMSA showing LexA_Pa_^S125A^ binding to SOS box dsDNA, in the presence of increasing concentrations of A12.

### A12 Does Not Impair LexA_Pa_ DNA-Binding Activity

LexA_Pa_ DNA-binding activity—which has to be preserved for an anti-SOS strategy to be effective—resides on the NTD but requires dimerization via the CTD. In this context, to rule out any interfering effect of A12 on LexA_Pa_ binding to SOS-box DNA, an EMSA was performed by incubating LexA_Pa_ with operator dsDNA sequences and different doses of A12, before analyzing the mixtures on native polyacrylamide gels. Even in the presence of a 60-fold molar excess of A12, LexA_Pa_ retained full SOS box DNA-binding activity, as confirmed by the conserved band shifts to a higher molecular weight (Figure 7C).

### A12 Preliminary Testing for Antimicrobial and Antivirulence Activity

Given the promising inhibitory activity of A12 on the SOS system in vitro, as well as the covalent reactivity of A12 (which may lead to targeting several different factors and pathways), we sought to evaluate its effect on *P. aeruginosa* cells and its interactions with known antimicrobial agents.

First, compound A12 alone showed no antimicrobial activity on *P. aeruginosa* PAO1 (ATCC 15692), up to the tested concentration of 32 μg/mL.

A12 was then assayed at fixed concentration (32 μg/mL, 162 μM) in combination with various antibiotics known to induce SOS stress signaling at different levels,^33^ including fluoroquinolones (ciprofloxacin, levofloxacin, and moxifloxacin), tetracycline, β-lactams (ceftriaxone), and aminoglycosides (gentamycin).

The combination with A12 either had no effect on the minimal inhibitory concentrations (MIC) of the tested antibiotics or resulted in a 2-fold increase in the case of fluoroquinolones, which are genotoxic agents that strongly induce the SOS response (Table 1).

**Table 1:** Antimicrobial Susceptibility Testing of A12, Common Antibiotics, and Combinations Thereof on *P. aeruginosa* PAO1

**Antimicrobials**	**MIC on *P. aeruginosa* PAO1** (μg/mL)	
**A12** (32 μg/mL)	**(−)**	**(+)**
A12	>32	
Ciprofloxacin	0.0312	0.0625
Levofloxacin	0.0625	0.125
Moxifloxacin	0.0312	0.0625
Tetracycline	4	4
Ceftriaxone	8	8
Gentamycin	2	2

If A12 covalent binding to RecA could affect its biological activities, one likely explanation for the observed antagonistic effect of A12 on fluoroquinolones might be the inhibition of RecA-dependent programmed cell death (PCD). Indeed, in *P. aeruginosa,* two distinct PCD pathways are regulated by the LexA-like autoproteolytic transcriptional repressors PrtR and AlpR and triggered by genotoxic damage via the RecA-induced autoproteolysis of these repressors.^10,11^ Mutations that prevented PrtR and AlpR autoproteolysis (which might be partially mimicked by A12-mediated impairment of RecA coprotease) have been demonstrated to reduce the susceptibility of *P. aeruginosa* to antibiotics, with MIC increases analogous to the ones that we registered in the presence of A12.^10^ However, we cannot rule out the involvement of other molecular mechanisms, given the complex regulatory network that characterizes *P. aeruginosa* response to stressors and that shapes its plasticity to environmental cues.^33,34^

Since stress-response pathways have been widely linked to virulence traits, including host tissue colonization,^11,35^ we next sought to determine whether A12 can reduce the binding of *P. aeruginosa* to lung epithelia cells and macrophages. The effect of A12 on *P. aeruginosa* intracellular growth was also assessed, as internalized bacteria often serve as a reservoir protected from antibiotics and immune defenses that hinders infection eradication.^36^ Figure 8 shows that the total (adherent + intracellular) burden of *P. aeruginosa* PAO1 on THP-1-derived macrophages but not on A549 lung cells is diminished upon treatment with either ciprofloxacin or A12. Their combination resulted in a significantly reduced colonization of A549 as well. Moreover, A12 synergized with ciprofloxacin to reduce the intracellular burden of PAO1 in both cell types. These results indicate that A12 could exert an antivirulence activity on *P. aeruginosa*, reducing its host colonization potential. Treatment with ciprofloxacin, A12 or their combination retained a cell viability higher than 80% compared to the untreated control on both cell lines (Figure S7), ruling out the possibility that the observed differences in *P. aeruginosa* colonization are due to a compromised integrity of host cells by the tested compounds. While promising, these observations require further investigations on A12 mechanism of action at molecular level.

**Figure 8:**
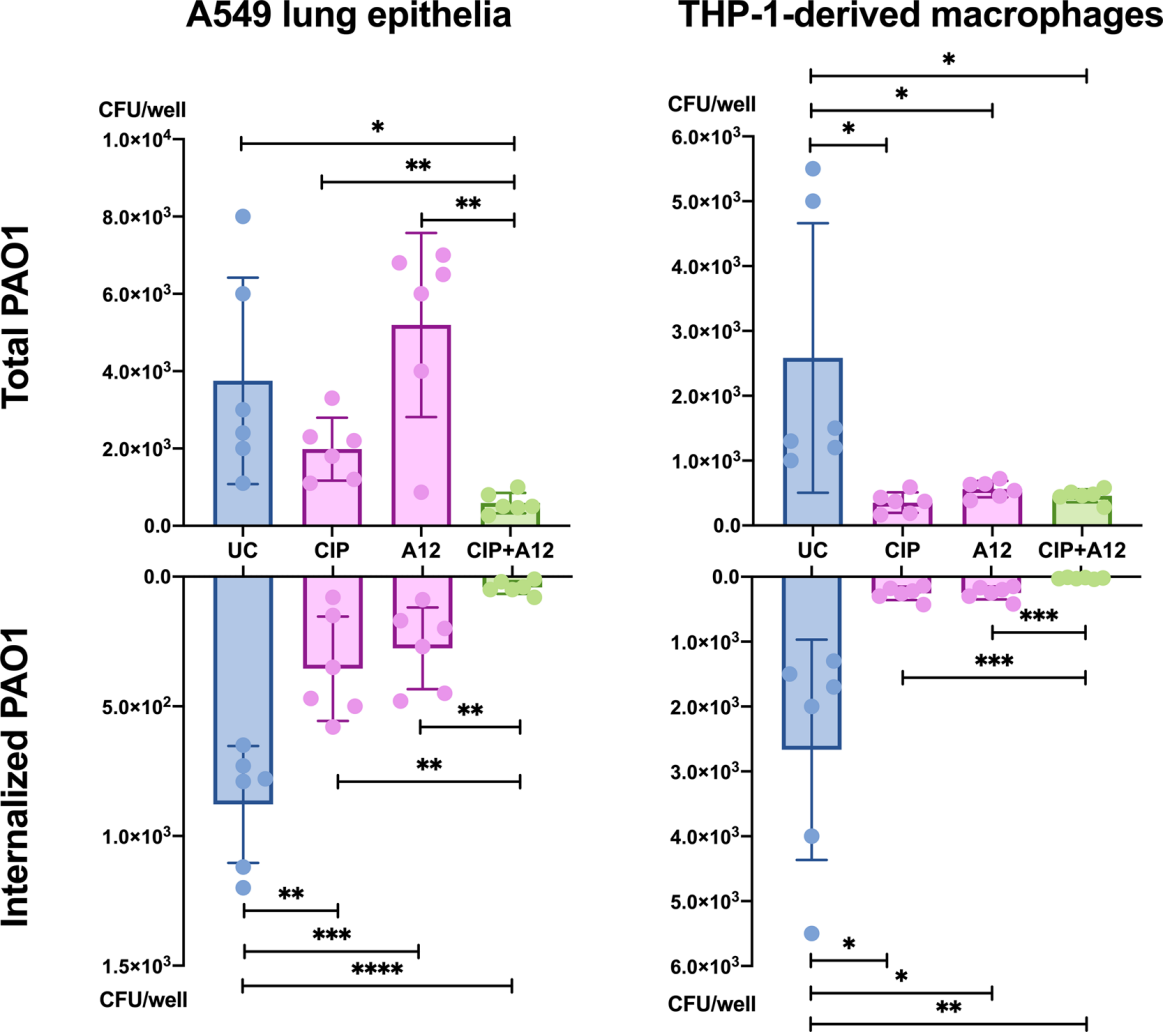
A12 inhibition of cell colonization by *P. aeruginosa*PAO1. Bacterial burdens (colony-forming units (CFU)/well) of total or intracellular (internalized) PAO1 on either A549 lung epithelial cells or THP-1-derived macrophages are displayed. Data represent averages of six replicates ±standard deviation. UC, untreated control (DMSO); CIP, ciprofloxacin (0.5 μg/mL); A12, A12 compound (100 μg/mL); CIP + A12, combination thereof. *P*-values of *t* tests: *p < 0.05, **p < 0.01, ***p < 0.001, ****p < 0.0001.

## Discussion

In the present work, potential inhibitors of the *P. aeruginosa* SOS system have been searched among small molecules by medium-throughput screening in vitro. One hit inhibitor of RecA_Pa_*-induced LexA_Pa_ autoproteolysis was discovered, and two different assays—relying on FP and SDS-PAGE to follow LexA_Pa_ self-cleavage—were used to characterize its dose–response relationship. Obtained IC_50_ values lie in the mid micromolar range, with differences in estimates attributed to intrinsic dissimilarities of the two techniques. In particular, different LexA_Pa_ variants (FlAsH-LexA_Pa_^CTD^ vs full-length LexA_Pa_) were used in the two assays, which might account for diverse affinities for A12. In the FP-based assay, the presence of thiol-reactive species, such as unreacted FlAsH-EDT_2_ and unlabeled 4Cys-tagged LexA_Pa_^CTD^, could account for partial A12 depletion and interference with the system. These species may form undesired covalent bonds with the A12 thiol group, likely competing with its inhibitory reaction on FlAsH-LexA_Pa_ and potentially leading to an underestimation of inhibitory activity.

The hit-to-lead optimization of A12 by targeted pharmacophore exploration would benefit from insights into the A12 mechanism of action. To this aim, A12 interaction with RecA_Pa_ and LexA_Pa_ underwent biochemical and biophysical investigation by means of DSF and ITC. The former technique revealed that A12 induced *T*_m_ variations on both the tested proteins, with a particular stabilizing effect on RecA_Pa_, a clear indication of binding.^37^ Conversely, increasing concentrations of A12 transformed the biphasic melting curve of LexA_Pa_ into a monophasic curve with a generally higher sensitivity to thermal denaturation. Biphasic melting curves are typical of proteins that either consist of two independently folding domains^37^ or assume a dimeric organization.^38^ Both cases could potentially apply to LexA_Pa_, as it is composed of two domains connected by a long and flexible linker and it behaves as a dimer in solution.^16^ Therefore, DSF data suggest that A12 could bind LexA_Pa_ in a region at the boundary of the C-terminal and N-terminal domains or close to the dimerization surface. On the other hand, ITC confirmed that A12 binds to both LexA_Pa_ and RecA_Pa_, with approximately 2 equivalent binding sites on each protein, but a higher affinity toward the transcriptional repressor LexA_Pa_ (*K*_D_^App^ of 7.4 μM) than for the coactivator RecA_Pa_ (*K*_D_^App^ of 18 μM).

A12 underwent MS analysis, showing that it mainly exists as a disulfide-bridged self-dimer in solution, reverting back to its synthesis intermediate. More notably, MS analysis evidenced that A12 binds RecA_Pa_ and LexA_Pa_ covalently, revealing a different binding mechanism on the two proteins: a condensation reaction in the former case and a disulfide bridging reaction in the latter one. Further activity testing in vitro, in the presence of a reducing agent, confirmed that the formation of disulfides with LexA_Pa_ is crucial for A12 activity. To confirm that the species mainly responsible for LexA binding and inhibition is the reduced A12 monomer, a molecule mimicking an A12 self-dimer devoid of thiols was tested and was inactive in a broad range of concentrations, excluding that the biologically active form could be represented by the dimeric A12 adduct. In light of such results, we hypothesize that the oxidized A12 dimer and its monomeric reduced form define an equilibrium in solution that limits the concentration of monomeric A12 available to bind and inhibit LexA_Pa_. This suggests that the affinity of A12 toward LexA_Pa_ might be higher than the calculated values and further explains the apparent 2-fold molar ratio required to reach half saturation in ITC.

MS analysis allowed to identify Cys104 as the A12 binding site on LexA_Pa_: such residue is located in the C-terminal autoproteolytic domain, at the basis of the cleavable loop and in close proximity to the LexA_Pa_ dimerization interface, as observable in the previously solved structure of LexA_Pa_ (PDB: 8B0V).^16^ This allowed us to suggest that A12’s mechanism of action could rely on the constraint of the LexA_Pa_ cleavable loop in an inactive conformation or the alteration of the LexA_Pa_ dimeric architecture. To rule out the possibility that A12 might destabilize the dimeric architecture of LexA_Pa_ required for binding to SOS box dsDNA, an EMSA was performed. The obtained results show that A12 treatment maintains the LexA_Pa_ DNA-binding activity. Besides validating A12 as a hit SOS inhibitor, these data revealed that Cys104 can be targeted as a site for blocking LexA_Pa_ autocleavage, while keeping the SOS pathway silenced.

Preliminary antimicrobial susceptibility testing of *P. aeruginosa* PAO1 to several antibiotic families in the presence of A12 showed either no alteration of MIC values or a 2-fold increase of MIC for fluoroquinolones. Such antagonism might arise either from the targeting of SOS-unrelated processes or from an impact of A12 on RecA coprotease activity on regulators of PCD pathways (i.e., PrtR and AlpR). In line with our observations, mutations that suppressed the autoproteolytic activity of PrtR and AlpR have been previously shown to reduce the sensitivity of *P. aeruginosa* PAO1 to various antibiotic classes.^10^

Moreover, as highlighted by Kohli and co-workers in their analysis of *E. coli* mutants spanning different levels of SOS activation, substantial enhancement of antimicrobial efficacy is likely achievable only through complete suppression of LexA autoproteolysis,^39^ which requires further medicinal chemistry improvement of A12.

However, given the involvement of the DNA-damage response in virulence traits, including host colonization, we tested the effect of A12 on *P. aeruginosa* binding and intracellular colonization of A549 lung cancer cells and THP-1-derived macrophages. Our results showed that A12 reduced the internalized bacterial growth on both cell lines and displayed synergistic activity with ciprofloxacin. A12, alone or in combination with ciprofloxacin, also reduced the total PAO1 burden (adherent and intracellular) on THP-1-derived macrophages and A549 cells, respectively. These observations, despite requiring further elucidation of A12 activity on *P. aeruginosa* physiology, underline its potentiality in antivirulence approaches.

In conclusion, the screening and validation campaign described here led to the identification of a new potential LexA_Pa_ targetable site and a molecular scaffold for SOS pharmacological suppression. Our findings open the way for further investigation and optimization of A12, to derive a potent and specific lead for future applications against the spreading of multiresistant *P. aeruginosa* infections.

## Materials and Methods

### Chemical Synthesis and Characterization of Compounds

#### General Procedures

Reagents and solvents were purchased from Sigma-Aldrich and used without further purification. The reaction course and purity of the synthesized compounds were monitored by TLC using aluminum plates precoated with Silica gel with F254 nm (Merck KGaA, Germany). Melting points were determined with a B-540 melting point analyzer (Büchi Corporation, USA). IR spectra (ν, cm^–1^) were recorded on a Perkin–Elmer Spectrum BX FT–IR spectrometer (Perkin–Elmer Inc., MA, USA) using KBr pellets. NMR spectra were recorded on a Bruker Avance III (400, 101 MHz) spectrometer (Bruker BioSpin AG, Switzerland). Chemical shifts were reported in δ ppm relative to tetramethylsilane (TMS) with the residual solvent as the internal reference ([D_6_]­DMSO, δ = 2.50 ppm for ^1^H and δ = 39.5 ppm for ^13^C). NMR data were reported as follows: chemical shift, multiplicity, coupling constant [Hz], integration, and assignment. Mass spectra were recorded on Waters SQ Detector 2 Spectrometer with an electrospray ionization (ESI) source. Elemental analyses (C, H, and N) were conducted using the Elemental Analyzer CE-440, and results were in good agreement (±0.3%) with the calculated values.

#### Synthesis of A12

2-Aminobenzenethiol was previously shown to react with α, β-unsaturated acids, producing benzothiazepine derivatives upon heating.^40,41^ 2-Aminobenzenethiol (**1**) was oxidized in dimethyl sulfoxide with atmospheric oxygen at 80 °C, producing 2-[(2-aminophenyl)­disulfanyl]­aniline (**2)**, which was then boiled for 13 h at reflux with acrylic acid in toluene. The mixture was cooled, and the formed crystals were filtered off. 3-[2-[[2-(2-Carboxyethylamino)­phenyl]­disulfanyl]­anilino]­propanoic acid (**3**) was purified by recrystallization from 2-propanol. A mixture of compound **3** (0.01 mol, 3.92 g), zinc dust (46 mmol, 3.00 g), 10% hydrochloric acid (20 mL), and 2-propanol (15 mL) was boiled for 15 min and filtered hot. Sodium acetate (0.5 g) was added to the mixture, and the formed crystals were filtered off and purified by recrystallization from 2-propanol, obtaining 3-(2-sulfanylanilino)­propanoic acid (compound **4**, referred to as A12 throughout this paper; Figure 9). Chemical characterization (^1^H NMR, ^13^C NMR, IR, and ESI-MS spectra) of compounds **3** and **4** is reported in Figures S3 and S4, respectively.

**Figure 9:**
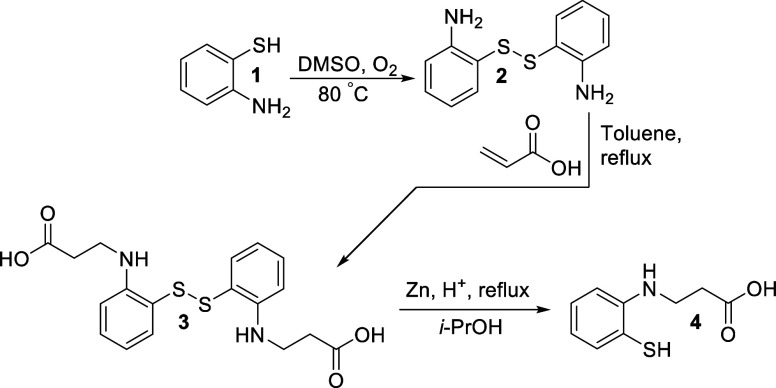
Chemical synthesis of A12 (compound 4).

##### 3-[2-[[2-(2-Carboxyethylamino)­phenyl]­disulfanyl]­anilino]­propanoic Acid (**3**); Figure S3

mp 117–118 °C.

^1^H NMR (400 MHz, DMSO-*d*_6_): δ 2.45 (4H, t, *J* = 6.8 Hz, 2NH*CH*_2_); 3.33 (4H, kv, *J* = 6.6 Hz, 2CH_2_CO); 5.42 (2H, t, *J* = 6.1 Hz, 2NH); 6.48 (2H, t, *J* = 7.4 Hz, H_Ar_); 6.68 (2H, d, *J* = 8.2 Hz, H_Ar_); 7.02 (2H, dd, *J* = 7.6; 1.5 Hz, H_Ar_); 7.13–7.29 (2H, m, H_Ar_); 12.30 (2H, s, 2OH).

^13^C NMR (101 MHz, DMSO-*d*_6_): δ 33.9 (2NH*CH*_2_); 39.2 (2CH_2_CO); 110.9, 116.4, 117.9, 132.4, 136.7, 149.2 (C_Ar_); 173.6 (2CO).

IR (KBr), ν­(cm^–1^): 3382, 3050 (2OH, 2NH); 1702 (2CO).

Calcd for C_18_H_20_N_2_O_4_S_2_, %: C, 55.08; H, 5.14; N,7.14. Found, %: C, 55.05; H, 5.03; N, 7.03.

C_18_H_20_N_2_O_4_S_2_, calcd exact mass = 392.09, MS (ESI^+^), 392.92 [M + H]^+^.

##### 3-(2-Sulfanylanilino)­propanoic Acid (**4**); Figure S4

mp 132–133.5 °C.

^1^H NMR (400 MHz, DMSO-*d*_6_): δ 2.46 (2H, t, *J* = 6.3 Hz, NHC*H*_2_); 2.97–3.17 (2H, m, CH_2_CO); 4.35 (1H, s, SH); 5.51 (1H, br s, NH); 6.59–6.73 (1H, m, H_Ar_); 6.75–6.90 (2H, m, H_Ar_); 7.28 (1H, d, *J* = 7.6 Hz, H_Ar_); 12.29 (1H, br s, OH).

^13^C NMR (101 MHz, DMSO-*d*_6_): δ 25.3 (NH*C*H_2_); 34.3 (*C*H_2_CO); 116.0; 117.4; 123.0; 132.8; 136.2; 145.2 (C_Ar_); 175.2 (CO).

IR (KBr), ν (cm^–1^): 3387 (OH); 3054 (NH); 1719 (CO).

Calcd for C_9_H_11_NO_2_S, %: C, 54.80; H, 5.62; N, 7.10. Found, %: C, 54.75; H, 5.57; N, 7.07.

C_9_H_11_NO_2_S, calcd exact mass = 197.05, MS (ESI^+^), 198.00 [M + H]^+^.

#### Synthesis of BG-191 and BG-194

Dicarboxylic acids **6** and **7** were obtained by the reaction of 2,2’-(ethane-1,2-diyl)­dianiline **(5)** with acrylic and crotonic acids, respectively (Figure 10).

**Figure 10:**
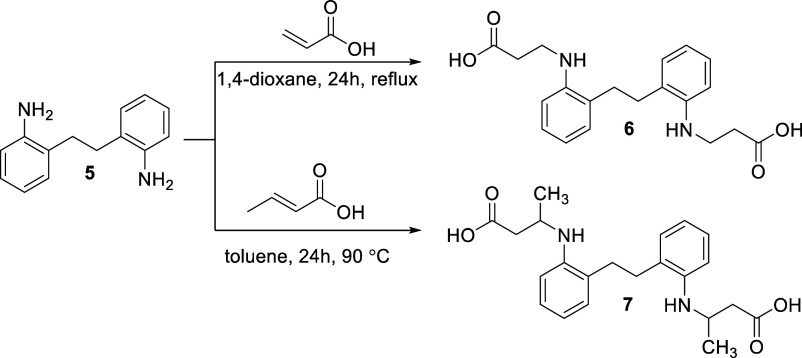
Chemical synthesis of BG-191 (compound 6) and BG-194 (compound 7).

A mixture of 2,2′-(ethane-1,2-diyl)­dianiline **(5)** (4.7 mmol, 1 g), acrylic acid (9.8 mmol, 0.71 g), and 1,4-dioxane (5 mL) was heated at reflux for 24 h. The mixture was cooled down, diluted with diethyl ether, and the formed crystals were filtered off. Compound **6** was purified by recrystallization from 2-propanol and water.

A mixture of 2,2′-(ethane-1,2-diyl)­dianiline **(5)** (4.7 mmol, 1 g), crotonic acid (14.1 mmol, 1.21 g), and toluene (15 mL) was heated at 90 °C for 24 h. The mixture was cooled down, 5% NaOH (20 mL) was added, and it was extracted with diethyl ether. The aqueous solution was acidified with acetic acid to pH 6, and the formed crystals were filtered off. Compound 7 was purified by recrystallization from 2-propanol. Chemical characterization (^1^H NMR, ^13^C NMR, IR, and ESI-MS spectra) of compounds **6** and **7** is reported in Figures S5 and S6, respectively.

##### 3,3′-((Ethane-1,2-diylbis­(2,1-phenylene))­bis­(azanediyl))­dipropionic Acid (**6**); Figure S5

mp 154–156 °C.

^1^H NMR (400 MHz, DMSO-*d*_6_): δ 2.54 (4H, t, *J* = 6.9 Hz, 2NH*CH*_2_); 2.66 (s, 4H, CH_2_CH_2_); 3,31 (4H, t, *J* = 6.9 Hz, 2CH_2_CO); 4.94 (2H, br s, 2NH); 6.46–6.67 (4H, m, H_Ar_); 6.98–7.15 (4H, m, H_Ar_); 12.03 (2H, s, 2OH).

^13^C NMR (101 MHz, DMSO-*d*_6_): δ 29.6 (2NH*CH*_2_); 33.6 (2CH_2_CO); 39.2 (CH_2_CH_2_); 109.7, 116.2, 126.1, 126.9, 128.8, 145.7 (C_Ar_); 173,7 (2CO).

IR (KBr), ν (cm^–1^): 3388, 3040 (2OH, 2NH); 1699 (2CO).

Calcd for C_20_H_24_N_2_O_4_, %: C, 67.40; H, 6.79; N, 7.86. Found, %: C, 67.17; H, 6.45; N, 7.54.

C_20_H_24_N_2_O_4_ calcd exact mass = 356.17, MS (ESI^+^), 356.91 [M + H]^+^.

##### 3,3′-((Ethane-1,2-diylbis­(2,1-phenylene))­bis­(azanediyl))­dibutyric Acid (**7**); Figure S6

mp 106–108 °C.

^1^H NMR (400 MHz, DMSO-*d*_6_): δ 1.18 (6H, d, *J* = 6.3 Hz, 2CH_3_); 3.24–2.37 and 2.51–2.60 (4H, 2m, 2CH_2_CO) 2.66 (s, 4H, CH_2_CH_2_); 3.83 (2H, q, *J* = 6.9 Hz, 2NH*CH*_2_); 5.54 (4H, br s, 2NH, 2OH); 6.45–6.61 (4H, m, H_Ar_); 6.94–7.10 (4H, m, H_Ar_).

^13^C NMR (101 MHz, DMSO-*d*_6_): δ 20.0 (2CH_3_); 29.7, 30.0 (2NH*CH*_2_); 41.1 (2CH_2_CO); 45.2 (CH_2_CH_2_); 110.2, 114.8, 116.0, 116.4, 125.3, 126.3, 126.5, 126.9, 128.9, 129.1, 144.9, 146.1 (C_Ar_); 173.9 (2CO).

IR (KBr), ν (cm^–1^): 3370, 2965 (2OH, 2NH); 1690 (2CO).

Calcd for C_22_H_28_N_2_O_4_, %: C, 68.73; H, 7.34; N, 7.29. Found, %: C, 68.52; H, 7.21; N, 7.11.

C_22_H_28_N_2_O_4_, calcd exact mass = 384.20, MS (ESI^+^), 384.94 [M + H]^+^.

#### Gene Cloning and Mutagenesis

All the plasmid vectors described in this work have been obtained as previously described.^16^ Briefly, the genomic DNA of *P. aeruginosa* ATCC 27853 was purified from an overnight liquid culture and used as the template to PCR-amplify the coding sequences of RecA_Pa_ (UniProtKB: P08280) and LexA_Pa_ (UniProtKB: P37452), using primer couples RecA_Pa_pColi.For/Rev and LexA_Pa.For/Rev (Supplementary, Table S1). The obtained amplicons were cloned in pColiExpressI (Canvax) and pETite C-His Kan vector (Lucigen), respectively, according to the manufacturer’s instructions, obtaining the plasmids pColiXP-RecA_Pa_ and pETite-LexA_Pa_.

The QuikChange Site-Directed Mutagenesis Kit protocol (Agilent Technologies) was applied to pETite-LexA_Pa_, using the mutagenic primers LexA_Pa_S125A.For and LexA_Pa_S125A.Rev (Supplementary, Table S1), obtaining the pETite-LexA_Pa_S125A plasmid vector.

The coding sequence of TetraCys-tagged LexA_Pa_ C-terminal domain (CTD) was amplified using primers LexA_Pa_CTD_4Cys.For and LexA_Pa_CTD_4Cys.Rev (Supplementary, Table S1) and cloned in pETite N-His SUMO Kan Vector (Lucigen) according to the manufacturer’s instructions, obtaining the plasmid pETite-SUMO-4Cys-LexA_Pa_^CTD^.

#### Recombinant Protein Expression and Purification

All the proteins (6His-RecA_Pa_, 6His-LexA_Pa_ wt and S125A mutant, and FlAsH-LexA_Pa_^CTD^) were recombinantly expressed in *E. coli* BL21­(DE3) cells, transformed with the appropriate plasmid vector, as previously reported.^16^ Briefly, 2 L cultures in LB broth were set up. Protein overexpression was induced by adding 1 mM IPTG to bacterial cultures in the late exponential growth phase (OD_600_: 0.6–0.8) and was carried out overnight at room temperature under vigorous shaking (180 rpm).

#### RecA_Pa_

Cells were harvested by centrifugation and resuspended in RecA_Pa_ Buffer A (10 mM Hepes, 300 mM NaCl, 10% v/v Glycerol, and 20 mM Imidazole, pH 8.0) supplemented with Protease Inhibitors Cocktail (SERVA). Bacterial cells lysis was performed by sonication. Cell debris was removed by centrifugation, and the lysate soluble fraction was loaded on a 5 mL HisTrap Excel IMAC column (Cytiva). After extensively washing the column with RecA_Pa_ Buffer A and with 50 mM imidazole in Buffer A, His-tagged RecA_Pa_ was eluted by linearly raising the imidazole concentration in the eluent from 50 to 500 mM in 3 column volumes.

IMAC fractions showing RecA_Pa_ as the main protein component in SDS-PAGE analysis were pooled together, concentrated using a Vivaspin Turbo Ultrafiltration unit (10 kDa MWCO; Sartorius), and buffer-exchanged to RecA_Pa_ Storage Buffer (10 mM Hepes, 300 mM NaCl, 10% Glycerol, 1 mM MgCl_2_, 1 mM DTT, pH 7.0) by a HiTrap Desalting column (Cytiva) before storage at −80 °C for future usage in in vitro assays.

#### LexA_Pa_ Wild-Type and S125A Uncleavable Mutant

The same protocol was used for recombinant expression and purification of wild-type and mutant LexA_Pa_ variants.

Cells were harvested by centrifugation and resuspended in LexA_Pa_ Lysis Buffer (20 mM Tris–HCl, 150 mM NaCl, 20 mM Imidazole, 10% v/v Glycerol, pH 7.5) supplemented with Protease Inhibitors Cocktail (SERVA), 500 U of benzonase nuclease (Merck), and 1.5 mM MgCl_2_. Bacterial cell lysis was performed by sonication, and the crude lysate was incubated for 30 min at 4 °C to allow benzonase-mediated DNA digestion. Cell debris was removed by centrifugation, and the lysate soluble fraction was loaded on a 1 mL HisTrap Excel IMAC column (Cytiva). After extensively washing the column with LexA_Pa_ Buffer A (20 mM Tris–HCl, 150 mM NaCl, 10% v/v Glycerol, pH 7.5) and with 20 mM imidazole in Buffer A, His-tagged LexA_Pa_ was eluted by linearly raising the imidazole concentration in the eluent from 20 mM to 500 mM in 10 column volumes.

IMAC fractions showing LexA_Pa_ as the main protein component by SDS-PAGE analysis were pooled together, concentrated using a Vivaspin Turbo Ultrafiltration unit (5 kDa MWCO; Sartorius), and buffer-exchanged to LexA_Pa_ Buffer A by a HiPrep 26/10 desalting column (Cytiva).

6His-LexA_Pa_ variants were stored at −80 °C for future usage in in vitro assays.

#### FlAsH-LexA_Pa_^CTD^

Cells were harvested by centrifugation and resuspended in FlAsH-LexA_Pa_ Lysis Buffer (20 mM Tris–HCl, 150 mM NaCl, 20 mM Imidazole, 10% v/v Glycerol, 0.1 mM DTT, pH 7.5) supplemented with Protease Inhibitors Cocktail (SERVA). Bacterial cell lysis was performed by sonication.

Cell debris was removed by centrifugation, and the lysate soluble fraction was loaded on a 1 mL HisTrap Excel IMAC column (Cytiva). After extensively washing the column with FlAsH-LexA_Pa_^CTD^ Buffer A (20 mM Tris–HCl, 150 mM NaCl, 10% v/v Glycerol, 0.1 mM DTT, pH 7.5) and with 20 mM imidazole in Buffer A, 6His-SUMO-TetraCys-LexA_Pa_^CTD^ was eluted by linearly raising the imidazole concentration in the eluent from 20 to 500 mM in 10 column volumes.

IMAC fractions showing 6His-SUMO-TetraCys-LexA_Pa_^CTD^ as the main protein component by SDS-PAGE analysis were pooled together, diluted three times in FlAsH-LexA_Pa_^CTD^ Buffer A (to reduce imidazole concentration below 150 mM), and supplemented by 1 mM DTT, 1 mM EDTA, 0.1% v/v NP-40, and an excess of Expresso Sumo Protease. Following a 2 h incubation at room temperature with gentle shaking, 100 μM FlAsH-EDT_2_ was added to the reaction mix, and the incubation was prolonged overnight at 4 °C. The mixture was first concentrated using a Vivaspin Turbo Ultrafiltration unit (5 kDa MWCO; Sartorius) and buffer-exchanged to FlAsH-LexA_Pa_^CTD^ Final Buffer (20 mM tris–HCl, 150 mM NaCl, 10% v/v Glycerol, pH 7.5) by a PD-10 desalting column (Cytiva). Then, to remove 6His-SUMO fragments and uncleaved protein constructs from the final sample, the mixture was passed through a 1 mL HisTrap Excel IMAC column (Cytiva), and the flowthrough was recovered. FlAsH-LexA_Pa_^CTD^ was stored at −80 °C for future usage in in vitro assays.

### RecA_Pa_ Activation

Biologically active RecA_Pa_/ssDNA/ATPγS nucleoprotein filaments (RecA_Pa_*) were produced by incubating 6His-RecA_Pa_ with SKBT25-18mer ssDNA ([RecA_Pa_]:[18mer] = 3.5:1) and 1 mM ATPγS overnight at 4 °C.

### FP-Based Library Screening and Hits Validation

A custom-made library of 318 chemical fragments (25 mg/mL stock solutions in DMSO), provided by P. Kavaliauskas (Weill Cornell Medicine of Cornell University)^30^ was screened in vitro for their ability to inhibit RecA_Pa_*-mediated LexA_Pa_ autoproteolysis by a Fluorescence Polarization (FP)-based assay.^20,21,29^ Twenty microliter reactions were set up in Nunc 384-Well Black microplates, and final reaction mixtures included 1 μM FlAsH-LexA_Pa_^CTD^, the compounds (5% v/v DMSO in all the samples, including the controls devoid of compounds), and 1 μM RecA_Pa_* (FP Buffer in the negative control; 30 mM Hepes, 150 mM NaCl, pH 7.1). The initial screening was performed with two dilutions of the compounds (25 and 125 μg/mL). The FP signal was measured after a 1 h incubation at 37 °C by an EnVision MultiMode Plate Reader (PerkinElmer) equipped with opportune FP filters and mirrors. To obtain LexA_Pa_^CTD^ autoproteolysis percent inhibition, FP data were scaled from 0% (corresponding to the positive control sample) to 100% (negative control). 5% concentration of DMSO had a minor effect on RecA_Pa_*-induced FlAsH-LexA_Pa_^CTD^ autoproteolysis, as shown in Figure S1A.

Compounds displaying an inhibition of at least 50% at the highest concentration and a rather consistent increase in activity at the two tested concentrations were selected as the hits.

The same FP assay was carried out on serial dilutions of selected compounds to determine the dose–response relationship. To evaluate the effect of a reducing environment, 2 mM Tris­(2-carboxyethyl)­phosphine (TCEP) was added to the reaction mixtures. The FP signal of FlAsH-LexA_Pa_^CTD^ alone was detected for 5 min before adding the compounds. The system was monitored for an additional 5 min in the presence of the tested compounds in order to observe potential aggregation events. Then, RecA_Pa_* was added, and the autoproteolysis reaction was followed for 1 h at 37 °C (1 reading per minute). FlAsH-LexA_Pa_^CTD^ autoproteolysis percent inhibition was calculated as mentioned above and plotted as a function of compound concentration. Experimental data fitting was performed with GraphPad Prism 8 to obtain the absolute IC_50_.

### RecA_Pa_*-Induced LexA_Pa_ Autoproteolysis Assay by SDS-PAGE

SDS-PAGE was used as an orthogonal technique to FP to validate the dose-dependent inhibition of RecA_Pa_*-induced LexA_Pa_ autocleavage by the selected hit compound.

Full-length LexA_Pa_ was used instead of FlAsH-LexA_Pa_^CTD^, but reaction volumes, molar concentrations of reagents, incubation time, and temperature were kept the same as in the FP-based assay. Following 1 h of incubation at 37 °C in the presence of RecA_Pa_*, reactions were stopped by adding Laemmli solubilization buffer and freezing the samples at −20 °C.

Samples were boiled 5 min at 95 °C before being loaded on 4–12% polyacrylamide gels. After Coomassie staining of the gels, the intensity of the bands corresponding to LexA_Pa_ and its autoproteolytic fragments was quantified using ImageJ. Data obtained from LexA_CTD/NTD_ bands densitometry were used to estimate LexA self-cleavage percent inhibition (scaling all the data from 0%, positive control, to 100%, negative control). Autocleavage inhibition data were plotted as a function of compound concentration and submitted to nonlinear regression in GraphPad Prism 8 to determine the absolute IC_50_.

### Electrophoretic Mobility Shift Assay

An EMSA of the SOS-box dsDNA in the presence of full-length LexA_Pa_S125A and different concentrations of the hit compound was performed to observe the potential interference effects of the selected drug on LexA_Pa_ binding to operator DNA sequences.

SOS-box dsDNA was produced by mixing equimolar amounts of AT-repeat_For and AT-repeat_Rev oligonucleotides (Supplementary, Table S1) in annealing buffer (10 mM Tris–HCl, 50 mM NaCl, 1 mM EDTA, pH 7.5), heating at 95 °C for 5 min, and then allowing the mixture to cool down to room temperature slowly overnight. The *E. coli* “AT-repeat” operator sequence^42^ was used as it is fully compatible with the *P. aeruginosa* SOS-box consensus (CTG-N_2_-T-N_7_-CAG).^9^

1 μM SOS-box dsDNA was incubated either alone or with 8 μM 6His-LexA_Pa_^S125A^ in EMSA buffer (50 mM Tris–HCl, 750 mM KCl, 0.5 mM EDTA, pH 7.4). Different concentrations of the tested compound were added to the mixtures (0, 50, 100, or 500 μM; 10% v/v DMSO in all the reactions, including controls) before a 40 min incubation at room temperature. Samples were treated with Purple DNA Loading Dye (New England Biolabs) and loaded on a NativePAGE 3–12% Polyacrylamide gel (Invitrogen). The gel was stained in SYBR Gold Nucleic Acid Stain (Invitrogen) for detecting DNA, then washed in deionized water and stained in SYPRO Ruby Protein Stain (Invitrogen) for detecting proteins. The obtained pictures were merged using ImageJ.

### Differential Scanning Fluorimetry

Differential Scanning Fluorimetry (DSF or “Thermofluor”) was performed on samples containing 4 μM RecA_Pa_ or LexA_Pa_^S125A^ in FP Buffer (30 mM Hepes, 150 mM NaCl, pH 7.1), different concentrations of the selected compound, and SyproOrange 8X (Life Technologies; a final concentration of 10% v/v DMSO was present in all the samples, including controls, and had a negligible effect on protein denaturation; Figure S1B).

Fluorescence of the SyproOrange dye was measured by a StepOne Real-Time PCR System (Applied Biosystems), while increasing the temperature from 25 to 95 °C (0.5 °C/min). Normalized fluorescence intensity curves were compared to observe macroscopic differences in the recorded melting profiles, while derivative curves were analyzed to find exact protein melting temperatures (*T*_m_, corresponding to derivative curve minimum). Melting curves were repeated twice, and duplicates were considered coherent if *T*_m_ differences were lower than 1 °C.

### Isothermal Titration Calorimetry

64 μM RecA_Pa_ and 58.5 μM LexA_Pa_^S125A^ were titrated with the selected compound (2.5 mM solution) using a Microcal PEAQ-ITC instrument (Malvern Panalytical) at 25 °C with a stirring rate of 750 rpm. Proteins and the ligand were dissolved in ITC Buffer (20 mM Tris–HCl, 150 mM NaCl, 5% v/v DMSO, pH 7.5). An initial 0.4 μL injection (excluded from data analysis) was followed by 24 injections of 1.5 μL with a spacing of 150 s between each addition. Blank experiments were performed by injecting the titrant compound in a protein-devoid ITC Buffer. Blank curves were subtracted from all of the experiments. Data were analyzed by using the MicroCal PEAQ-ITC Evaluation software (Malvern Panalytical). Integrated heat signals were fitted by a “one set of sites” binding model to obtain enthalpy changes (Δ*H*), dissociation constants (*K*_D_), and stoichiometry of binding.

### Mass Spectrometry Analysis

MS analysis was carried out on 5 μL samples of compound A12 (2 mM), of the proteins 6His-LexA_Pa_ and 6His-RecA_Pa_ (50 μM), and of the same proteins incubated with 2 mM A12 (2 h incubation at 25 °C, 10% v/v DMSO in 100 mM NaHCO_3_ pH 7.0). Aliquots of the latter samples were treated with 5 mM dithiothreitol (DTT) for 1 h at 25 °C before being buffer-exchanged in 100 mM NaHCO_3_ at pH 7.0. Samples were analyzed by an electrospray ionization (ESI) mass spectrometer equipped with a Xevo G2-XS ESI-Q-TOF mass spectrometer (Waters Corporation, Milford, Massachusetts, USA). Measurements were carried out at 1.5–1.8 kV capillary voltage and 30–40 V cone voltage.

Tryptic digestion was obtained at a 1:20 trypsin to protein ratio (by weight) after the incubation of the proteins (6His-LexA_Pa_ and 6His-RecA_Pa_) with chemical compound A12. The reaction was left at 37 °C overnight and then stopped by freezing at – 20 °C. Proteolytic mixtures were analyzed by LC–MS, using an Agilent AdvanceBio Peptide Map column (2.1 × 150 mm × 2.7 μm; Santa Clara, CA, USA) connected to an Acquity H-Class instrument (Waters Corporation, Milford, Massachusetts, USA). The elution was performed at a flow of 0.2 mL/min with the following acetonitrile/0.1% formic acid–water/0.1% formic acid gradient: 2–65%, 36 min, 65–98%, 2 min. Mass analyses were carried out with the same capillary voltage and cone voltage described before.

### Antimicrobial Susceptibility Testing

The MIC of compound A12 and antibiotics was determined by using the Clinical Laboratory Standard Institute (CLSI) recommendations with minor modifications. The MIC determination assay was performed in 96-well plates, and the tested compounds were prepared in 2-fold serial dilutions (0.062–32 μg/mL) as 2X stock solutions in Cation-adjusted Mueller Hinton Broth (CAMBH) supplemented with 2% of DMSO (100 μL/well). The *P. aeruginosa* PAO1 (ATCC 15692) inoculum was prepared by diluting an overnight culture 1:5 with fresh CAMBH and further incubating it at 37 °C for 2–4 h to reach OD_600_ 0.5 (approximately 2 × 10^8^ CFU/ml). Then the culture was further diluted in fresh CAMBH to achieve 5 × 10^5^ CFU/mL, and inoculum (100 μL) was transferred to 96-wells, achieving the final concentration of inoculum and compounds (as well as 1% DMSO). Microplates were incubated in a humidified incubator for 18 h at 37 °C, and the bacterial growth was quantified by OD_600_. According to CLSI, MICs are defined as the lowest concentrations of antimicrobial agents that prevent visible growth.

### Cell Lines and Culture Conditions

Human A549 type II pneumocytes (ATCC CCL-185) and human THP-1 monocytes (ATCC TIB-202) were obtained from the American Type Culture Collection. A549 cells were maintained in DMEM/F12 medium supplemented with GlutaMAX, PenStrep, and 10% heat-inactivated FBS. THP-1 monocytes were maintained in complete RPMI medium supplemented with GlutaMAX and 10% heat-inactivated FBS. Both cell lines were cultured at 37 °C with 5% CO2 and subcultured when they reached confluency.

THP-1-derived macrophages were generated from THP-1 monocytes as previously described, with minor modifications.^43,44^ Briefly, THP-1 monocytes were washed three times with antibiotic-free RPMI 1640 medium and resuspended in complete antibiotic-free RPMI 1640 supplemented with 300 nM phorbol 12-myristate 13-acetate (PMA). Cells were incubated for 24 h, followed by a 48 h rest period in PMA-free RPMI 1640 prior to use in subsequent experiments.

### *P. aeruginosa* PAO1 Tissue Culture Infections

An overnight culture of *P. aeruginosa* PAO1 in tryptic soy broth (TSB) was diluted 1:100 in fresh TSB and incubated at 37 °C with shaking (150 rpm) until an OD_600_ of 0.5. Cells were harvested by centrifugation (5000*g* for 10 min) and washed three times with antibiotic-free DMEM supplemented with FBS (for A549 cells) or antibiotic-free RPMI supplemented with FBS (for THP-1-derived macrophages), each containing 0.2% DMSO. The inoculum was adjusted to approximately 2.5 × 10^5^ CFU/mL in the respective antibiotic-free medium containing DMSO. Ciprofloxacin (0.5 μg/mL), A12 (100 μg/mL), or their combination (0.5 μg/mL ciprofloxacin and 100 μg/mL A12) were added to the adjusted inoculum, and cultures were incubated at 37 °C for 10 min.

One day prior to infection, A549 cells were trypsinized using trypsin–EDTA and seeded into 24-well plates at a density of 5 × 10^5^ cells/well in complete, antibiotic-free DMEM supplemented with FBS. PMA-differentiated THP-1-derived macrophages were similarly trypsinized and seeded into 24-well plates at 5 × 10^5^ cells/well in complete, antibiotic-free RPMI supplemented with FBS. Cells were incubated overnight to allow for attachment. Immediately prior to infection, the medium was aspirated, and cells were washed three times with Dulbecco’s phosphate-buffered saline. Ciprofloxacin-, A12-, or combination-treated *P. aeruginosa* inocula were then added to the cells (1 mL per well, leading to a PAO1/host cell MOI of 0.5). Infection was synchronized by centrifuging plates at 1000*g* for 1 min. The infected plates were subsequently incubated at 37 °C in a 5% CO_2_ atmosphere for 3 h. Following the infection period, the medium was aspirated, and cells were washed three times with DPBS to remove unattached bacteria and residual test compounds.

For total *P. aeruginosa* PAO1 enumeration (adherent + intracellular), 1 mL of lysis solution (0.5% Triton X-100 in DPBS) was added to each well, and the plates were incubated for 10 min at room temperature before scraping using sterile microscrapers.

For intracellular *P. aeruginosa* PAO1 enumeration, complete tissue culture medium containing 200 μg/mL gentamicin was incubated in each well for 2 h (37 °C, 5% CO_2_ atmosphere) to eliminate extracellular bacteria before proceeding to cell lysis as described above.

In both cases, the resulting lysates were serially diluted in DPBS and plated on tryptic soy agar plates. Plates were incubated at 37 °C for 24 h, after which colonies were enumerated. Bacterial burden was expressed as the CFU per well.

### Cytotoxicity Assessment by MTT Assay

A549 cells were seeded in flat-bottom 96-well plates at 5 × 10^4^ cells/well. THP-1 monocytes were differentiated into macrophages using PMA as described above, rested in PMA-free medium, detached by trypsinization, and seeded at 5 × 10^4^ cells/well. Plates were incubated overnight to allow cell attachment. The following day, medium was replaced with test medium containing ciprofloxacin (0.5 μg/mL), A12 (100 μg/mL), their combination (0.5 μg/mL ciprofloxacin and 100 μg/mL A12), SDS (0.5% w/v), or DMSO (0.2%, “untreated control”, UC), and cells were incubated for 3 h. Test medium was then removed and replaced with fresh medium containing 0.5 mg/mL 3-(4,5-dimethylthiazol-2-yl)-2,5-diphenyltetrazolium bromide (MTT). After a 2 h incubation, the medium was discarded, formazan crystals were solubilized with 100 μL of DMSO, and absorbance was measured at 570 nm using a spectrophotometer (Varioskan LUX Multimode Microplate Reader, Thermo Fisher Scientific). Cell viability was expressed as a percentage relative to the untreated controls.

## Supplementary Material



## Notes

Oligonucleotides sequences and description; control experiments and false positive hit assessment; MS data; chemical characterization of compounds ^1^H NMR, ^13^C NMR, IR, and ESI-MS spectra; and cell viability control experiments (PDF)
