# Supplementary material for: Identification and Characterization of a Small-Molecule Inhibitor of the Pseudomonas aeruginosa SOS Response
Source: ACS Infect Dis. 2025 Nov 7;11(12):3465–80. doi: 10.1021/acsinfecdis.5c00467 (PMC12706794; doi:10.1021/acsinfecdis.5c00467)

SUPPORTING INFORMATION

Identification and characterization of a small-molecule inhibitor of the *Pseudomonas aeruginosa* SOS response

Filippo Vascon[1], Benedetta Fongaro[2], Vytautas Mickevičius[3], Antonella Pasquato[4], Birute Grybaite[3], Vidmantas Petraitis[5], Rahma Ben Abderrazek[6], Patrizia Polverino de Laureto[2], Donatella Tondi[7], Povilas Kavaliauskas[3,8] and Laura Cendron[1,*]

[1] Department of Biology, University of Padova, Via Ugo Bassi 58/b, 35131 Padova, Italy.

[2] Department of Pharmaceutical and Pharmacological Sciences, University of Padova, via F. Marzolo 5, 35131 Padova, Italy.

[3] Department of Organic Chemistry, Kaunas University of Technology, Radvilenu Rd. 19, LT-50254 Kaunas, Lithuania

[4] Department of Surgery, Oncology and Gastroenterology, University of Padova, Via Giustiniani 2, 35128 Padova, Italy.

[5] Center for Discovery and Innovation, Hackensack Meridian Health, Nutley, New Jersey, United States of America.

[6] Laboratoire des Biomolécules, Venins et Applications Théranostiques, Institut Pasteur Tunis, Université Tunis El Manar, 13 Place Pasteur, Tunis, Tunisia.

[7] Department of Life Sciences, University of Modena and Reggio Emilia, Via Campi 103, 41125 Modena (MO), Italy.

[8] Department of Microbiology and Immunology, University of Maryland School of Medicine, Baltimore, Maryland, USA.

* Corresponding Author: Laura Cendron (laura.cendron@unipd.it)

Contents:

Table S1: Oligonucleotides

Figure S1: Assessment of DMSO effect on *in vitro* assays and characterization of compound E17 by the FP-based LexA autoproteolysis assay.

Figure S2: MS analysis of A12-modified $RecA_{Pa}$ and $LexA_{Pa}$

Figure S3: Characterization of compound **3** after chemical synthesis (^{1}H-NMR, ^{13}C-NMR, IR and ESI-MS spectra)

Figure S4: Characterization of compound **4** (A12) after chemical synthesis (^{1}H-NMR, ^{13}C-NMR, IR and ESI-MS spectra)

Figure S5: Characterization of compound **6** (BG-191) after chemical synthesis (^{1}H-NMR, ^{13}C-NMR, IR and ESI-MS spectra)

Figure S6: Characterization of compound **7** (BG-194) after chemical synthesis (^{1}H-NMR, ^{13}C-NMR, IR and ESI-MS spectra)

Figure S7: Measurement of A549 and THP-1-derived macrophages viability upon treatment with ciprofloxacin and A12.

Table S1: Oligonucleotides

Name	Sequence (5’-3’)	Notes
RecA_Pa.For	*ACCACCACCACAAGCTT***GAAAACCTGTATTTTCAGGGA**GACGAGAACAAGAAGCGCG	*N*=pColiXP homol. sequence; **N**=TEV cleavage site CDS;<br>N=CDS of $RecA^{Pa}$
RecA_Pa.Rev	*CTAATTAGGATCCGATT*CAATCGGCTTCGGCG	*N*=pColiXP homol. sequence;<br>N=CDS of $RecA^{Pa}$
LexA_Pa_CTD_4Cys.For	*CGCGAACAGATTGGAGGT***GGCTCTTGCTGTCCGGGTTGCTGC**GGCCTGCCGGTGATC	*N=pETite-SUMO homol. Seq.;*<br>**N**=tetraCys Tag (GSCCPGCC) CDS;<br>N=CDS of $LexA_{Pa}^{CTD}$ CDS
LexA_Pa_CTD_4Cys.Rev	*GTGGCGGCCGCTCTATTA*TCAGCGCCGGATCAC	*N=pETite-SUMO homol. Seq.;*<br>N=$LexA_{Pa}^{CTD}$ CDS
LexA_Pa.For	*GAAGGAGATATACATATG***CACCACCACCACCACCAC**GGTCAGAAGCTGACGCCCC	*N*=pETite C-His homol. sequence;<br>**N**=6xHisTag CDS;<br>N=CDS of $LexA_{Pa}$
LexA_Pa.Rev	*GTGATGGTGGTGATGATG***TCA**GCGCCGGATCAC	*N*= pETite C-His homol. sequence;<br>**N**=stop codon<br>N=CDS of $LexA_{Pa}$
LexA_Pa_S125A.For	GCGTGCGCGGCATG**GC**CATGAAGGACATCGG	**N**=mutations
LexA_Pa_S125A.Rev	CCGATGTCCTTCATG**GC**CATGCCGCGCACGC	**N**=mutations
AT-repeat_For	GATGCCTGCGGATACTGTATATATATACAGTATCAATTCTGGCT	Annealing with AT-repeat_Rev to obtain SOS-box dsDNA
AT-repeat_Rev	AGCCAGAATTGATACTGTATATATATACAGTATCCGCAGGCATC	Annealing with AT-repeat_For to obtain SOS-box dsDNA
SKBT25-18mer	GCGTGTGTGGTGGTGTGC	Activation of $RecA_{Pa}$ for inducing $LexA_{Pa}$ autoproteolysis in functional assays

Figure S1: Assessment of DMSO effect on *in vitro* assays and characterization of compound E17 by the FP-based LexA autoproteolysis assay. (A) FP-based $RecA_{Pa}$*-induced FlasH-$LexA_{Pa}^{CTD}$ autoproteolysis assay showing that 5% v/v DMSO has a minor effect on LexA self-cleavage. (B) Thermal shift assay of $RecA_{Pa}$ and $LexA_{Pa}^{S125A}$ in the presence of 10% v/v DMSO, showing negligible effects on proteins melting behavior. (C) FP-based $RecA_{Pa}$*-induced FlasH-$LexA_{Pa}^{CTD}$ autoproteolysis assay in the presence of various concentrations of the false-positive hit E17, displaying high spikes likely due to unspecific aggregation events. (D) Dose-response curve of E17 on $LexA_{Pa}$ self-cleavage, as derived from normalization on data reported in panel C.

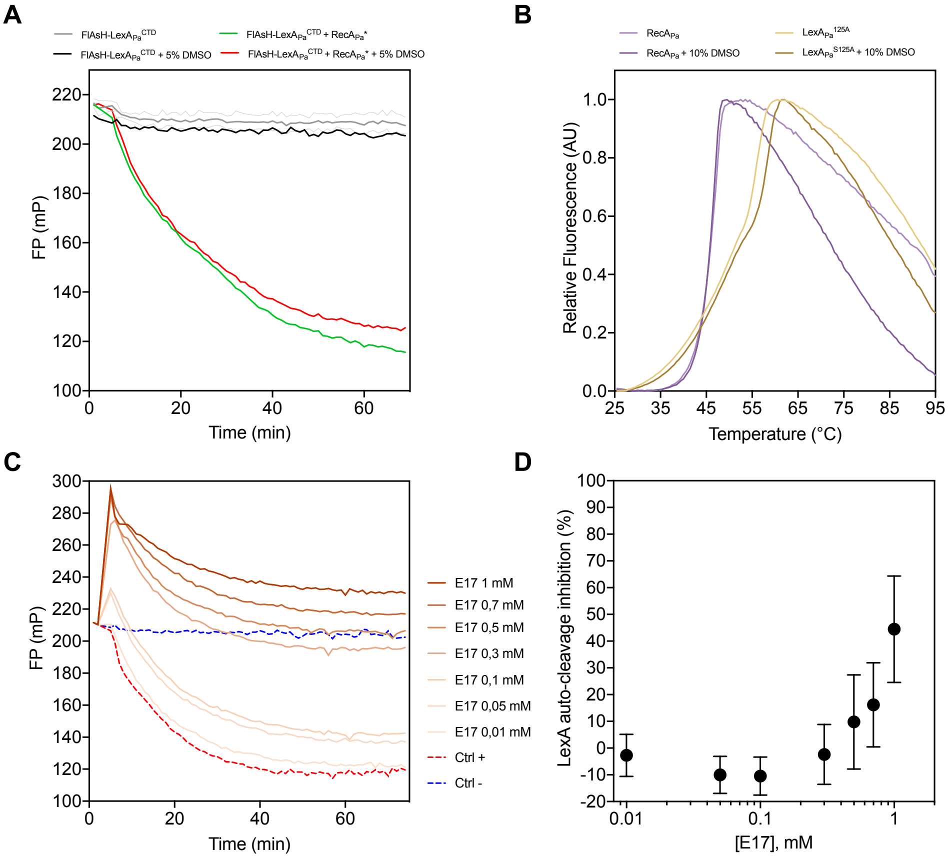

Figure S2: MS analysis of A12-modified $RecA_{Pa}$ and $LexA_{Pa}$. (A) Protein molecular masses found from MS analysis of the indicated samples. Molecular masses of found modified peptides in trypsin digestions are reported as well. Charge distributions of the chemically modified peptides of $RecA_{Pa}$-A12 (B) and $LexA_{Pa}$-A12 (C).

A

Sample	Fig.	Found Mass (Da)	Calculated Mass (Da)	Corresponding species
**$RecA_{Pa}$**	4A	38795.26 ± 0.08 (red)	38795.31	6His-$RecA_{Pa}$
**$RecA_{Pa}$-A12**	4B	38795.35 ± 0.10 (red)	38795.31	6His-$RecA_{Pa}$
		38974.23 ± 0.17 (blue)	38974.31	6His-$RecA_{Pa}$ + 179 Da
	S2B (Trypsin digestion)	1861.94 ± 0.31	1861.95	Fragment 285-300 + 179 Da
**$LexA_{Pa}$**	4A	23371.29 ± 0.20 (red)	23371.30	6His-$LexA_{Pa}$
**$LexA_{Pa}$-A12**	4B	23371.25 ± 0.20 (red)	23371.30	6His-$LexA_{Pa}$
		23566.85 ± 0.15 (blue)	23566.30	6His-$LexA_{Pa}$ + 195 Da
	S2C (Trypsin digestion)	2064.92 ± 0.07	2064.93	Fragment 95-112 + 195 Da
**$LexA_{Pa}$-A12+DTT**	4C	23371.49 ± 0.20 (red)	23371.30	6His-$LexA_{Pa}$

B

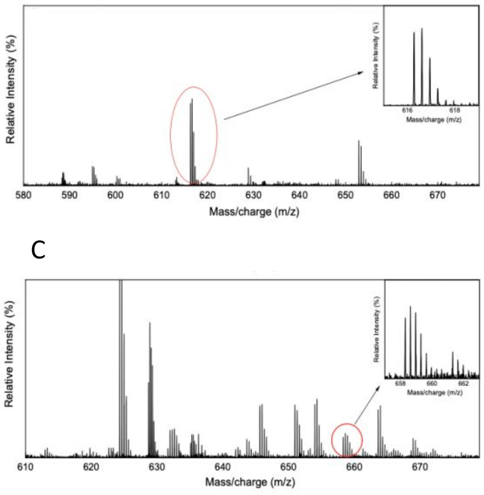

Figure S3: Characterization of compound 3 after chemical synthesis (^{1}H-NMR, ^{13}C-NMR IR and ESI-MS spectra)

^{1}H-NMR

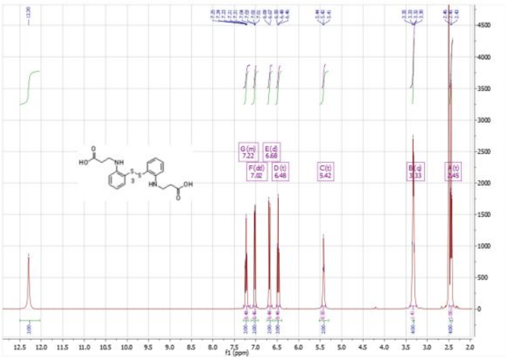

^{13}C-NMR

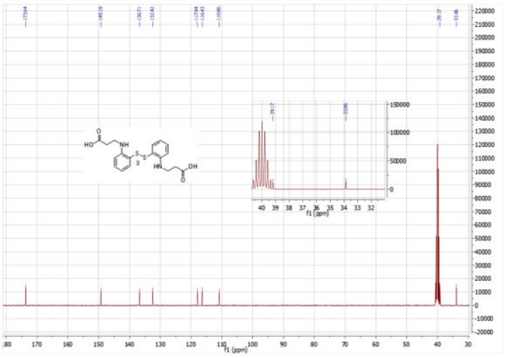

IR

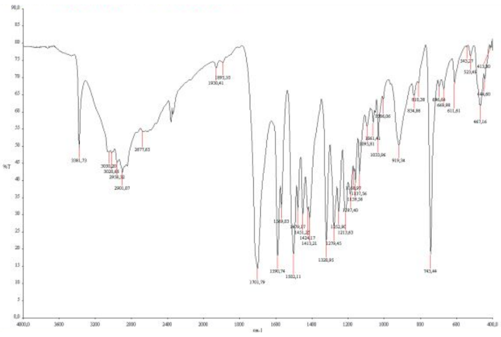

ESI-MS

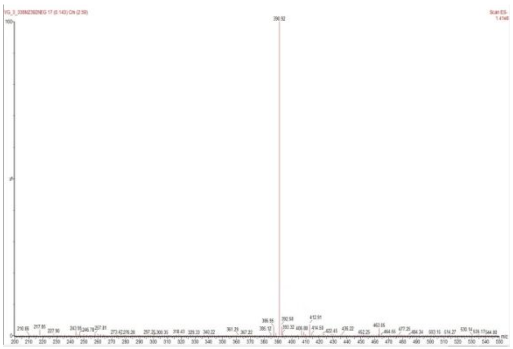

Figure S4: Characterization of compound 4 (A12) after chemical synthesis (^{1}H-NMR, ^{13}C-NMR IR and ESI-MS spectra)

^{1}H-NMR

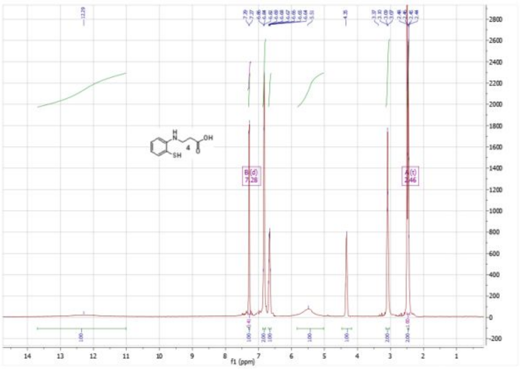

^{13}C-NMR

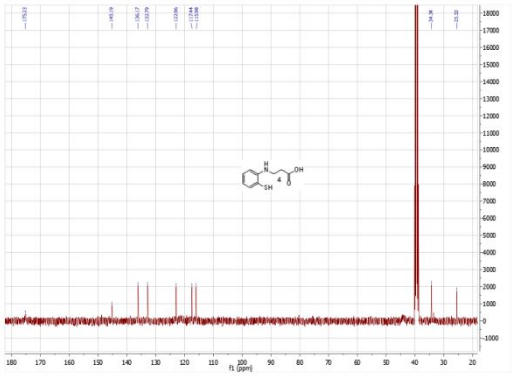

IR

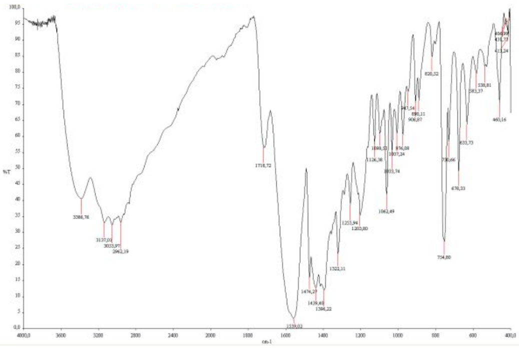

ESI-MS

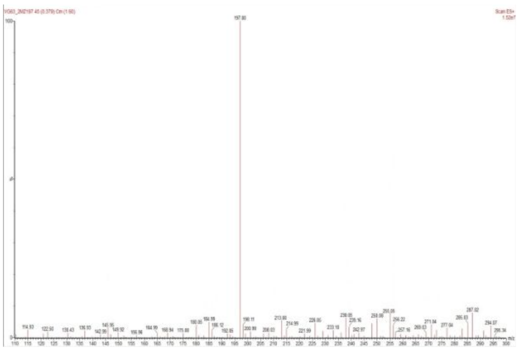

Figure S5: Characterization of compound 6 (BG-191) after chemical synthesis (^{1}H-NMR, ^{13}C-NMR IR and ESI-MS spectra)

^{1}H-NMR

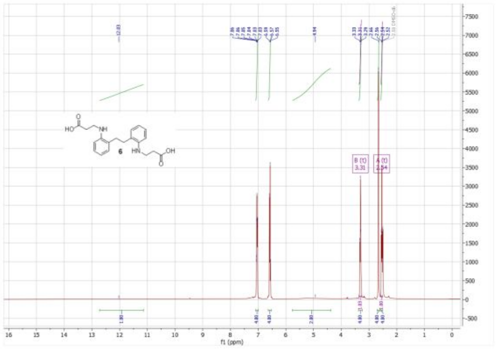

^{13}C-NMR

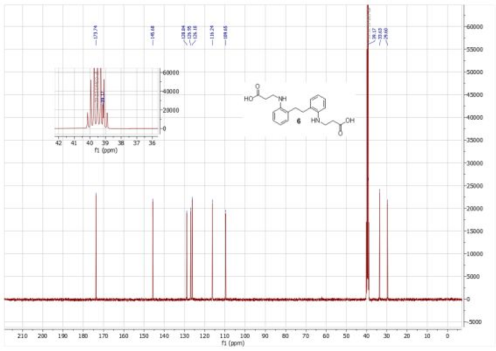

IR

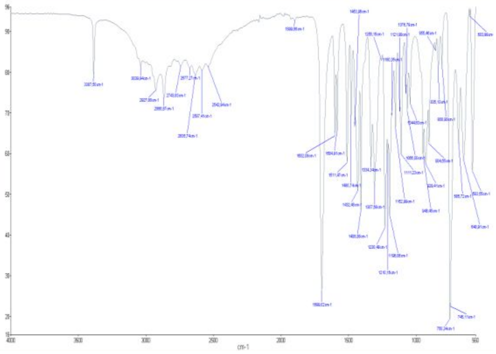

ESI-MS

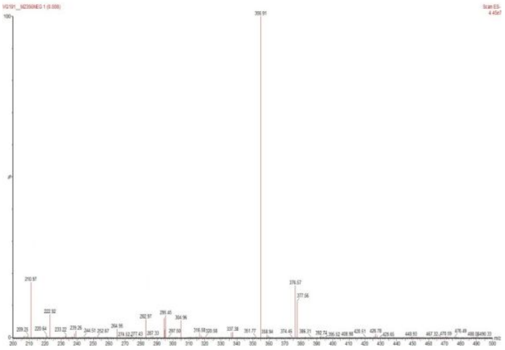

Figure S6: Characterization of compound 7 (BG-194) after chemical synthesis (^{1}H-NMR, ^{13}C-NMR IR and ESI-MS spectra)

^{1}H-NMR

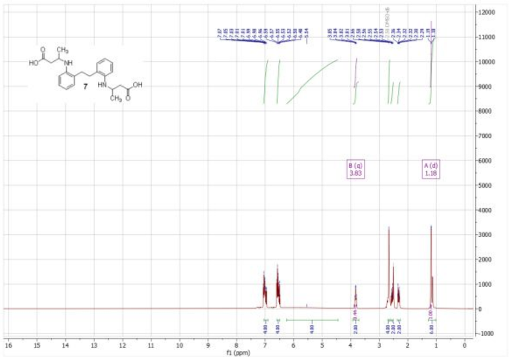

^{13}C-NMR

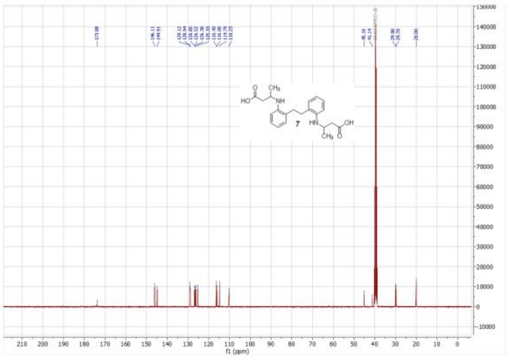

IR

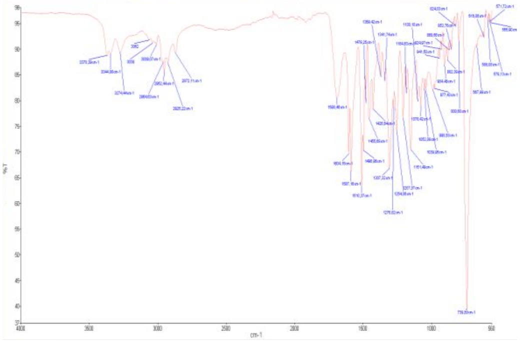

ESI-MS

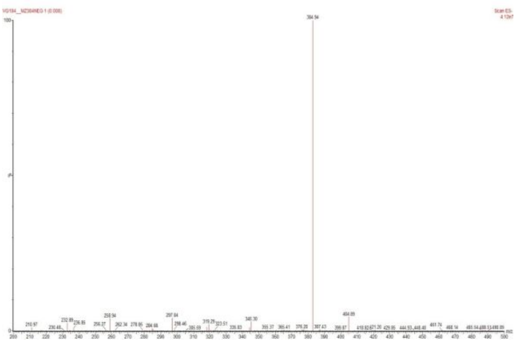

Figure S7: Measurement of A549 and THP-1-derived macrophages viability upon treatment with ciprofloxacin and A12. Treated samples were compared by t-tests to the untreated control (UC). P-values: * p<0.05, ** p<0.01, *** p<0.001, **** p<0.0001.

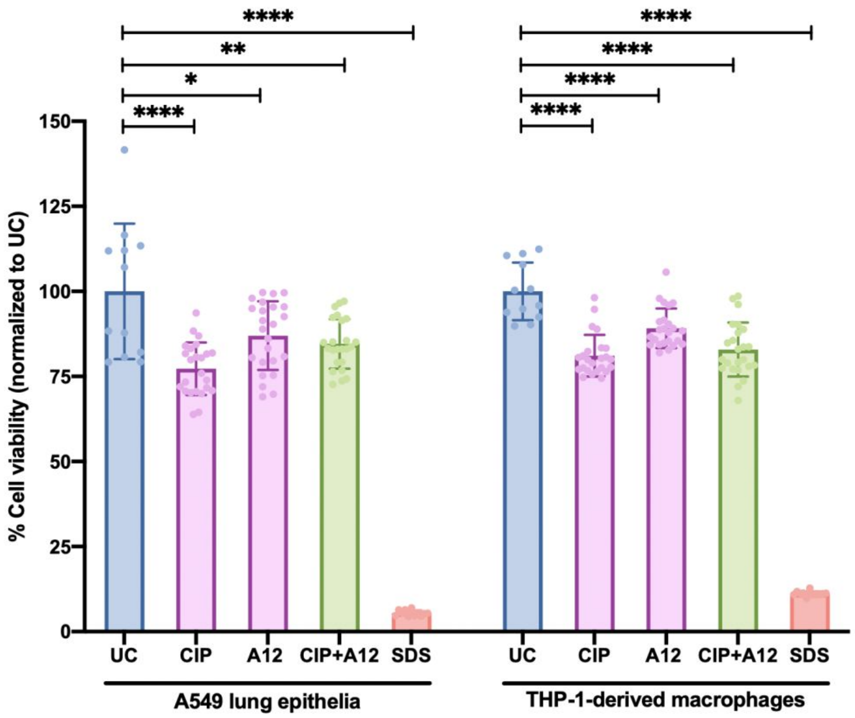

Supplement: Supplementary file 1 [file id5c00467_si_001.pdf]
